# Uncovering Novel Atrial Fibrillation Genetics Through Pleiotropic Overlap with Life’s Essential 8

**DOI:** 10.3390/biomedicines14061179

**Published:** 2026-05-22

**Authors:** Jingxian Wu, Xueying Qin, Shuting Xie, Liuyan Zheng, Huan Yu, Huairong Wang, Yalin Chen, Teng Li, Tao Wu, Dafang Chen, Yonghua Hu, Yiqun Wu

**Affiliations:** 1 Department of Epidemiology and Biostatistics, School of Public Health, Peking University, Beijing 100191, China; 2411210075@bjmu.edu.cn (J.W.); xueyingqin@bjmu.edu.cn (X.Q.); 2010306132@stu.pku.edu.cn (S.X.); zly101759@163.com (L.Z.); yuhh@pku.edu.cn (H.Y.); 1910306129@pku.edu.cn (H.W.); yalin@bjmu.edu.cn (Y.C.); tyylia@163.com (T.L.); twu@bjmu.edu.cn (T.W.); dafangchen@bjmu.edu.cn (D.C.); yhhu@bjmu.edu.cn (Y.H.); 2 Key Laboratory of Epidemiology of Major Diseases (Peking University), Ministry of Education, Beijing 100191, China

**Keywords:** atrial fibrillation, life’s Essential 8, pleiotropy, genetic susceptibility, gene

## Abstract

**Background/Objectives**: Atrial fibrillation (AF) is a complex polygenic disorder; its genetic architecture remains challenging to fully elucidate. **Methods**: In this study, we leveraged the extensive genetic overlap between AF and a spectrum of cardiometabolic and behavioral factors—collectively defined by Life’s Essential 8 (LE8)—to advance our understanding of its etiology. **Results**: We first estimated significant genetic correlations between AF and all LE8 components (r_g_: −0.11 to 0.19) using LD score regression. We then applied conditional false discovery rate analysis and detected 970 pleiotropic loci associated with AF and at least one LE8 trait. Subsequent colocalization analysis identified 179 loci harboring shared causal variants between AF and one or more LE8 components, which were further refined into 137 distinct colocalized regions. Through region-based annotation and functional predictors, we finally prioritized 164 candidate genes from these colocalized loci, including 40 novel genes. These candidate genes were enriched in pathways related to heart development and regulation of cardiac contraction, and were also enriched among molecular targets of otological agents. Among all LE8 components, blood pressure demonstrated the most extensive shared genetic architecture with AF, supported by the strongest genetic correlation, highest pleiotropic enrichment, and the greatest number of colocalized loci with AF. Polygenic risk scores constructed from these colocalized loci demonstrated significant associations not only for AF but also for arrhythmia and heart failure. **Conclusions**: Our findings establish a genetic pleiotropy-informed framework that enhances the discovery of novel risk loci of AF and advances our understanding of the shared genetic architecture and potential biological mechanisms between AF and LE8 components.

## 1. Introduction

Atrial fibrillation (AF), the most common clinically significant arrhythmia, poses a growing burden on patients [1,2,3] and healthcare systems [4,5,6] worldwide. As a complex polygenic disease, AF exhibits considerable heritability, with genetic factors contributing up to 62.1% of disease risk [7]. Early genetic studies, including linkage studies [8,9], candidate-gene approaches [10], and subsequent genome-wide association studies (GWASs) [11,12,13,14,15,16,17,18,19,20,21,22,23], have identified numerous risk loci, implicating ion channel dysfunction, developmental programs and structural remodeling, and cytoskeletal interactions in AF etiology [24]. Complementary GWASs on electrocardiographic traits [15,20] and multi-omics integrative analyses [25,26] further support the polygenic nature of AF. Two recent large-scale trans-ethnic GWASs have markedly expanded this genetic landscape, one integrating genome and exome sequencing data from 52,416 AF cases, highlighting the contribution of rare coding and structural variants [27]; and another that analyzed 181,446 AF cases, identifying genes involved in muscle contractility, cardiac development, and cell–cell communication [28]. To date, more than 300 risk loci have been identified for AF, yet collectively they explain only one fifth of its heritability [29], underscoring the need to further elucidate the genetic architecture of AF.

Emerging evidence indicates that trait-associated loci span more than half of the genome, with approximately 90% exhibiting pleiotropic effects [30]. This pervasive pleiotropy not only enhances our understanding of shared biological pathways underlying trait associations but also offers an innovative strategy to identify susceptibility loci that may be overlooked by conventional GWAS [31]. For instance, a pleiotropy-informed conditional false discovery rate (condFDR) approach has improved gene discovery in schizophrenia by leveraging genetic associations with cardiovascular disease risk factors, revealing additional susceptibility genes and providing mechanistic insights into their comorbidity [32]. Building on this concept, recent methodological advances continue to leverage pleiotropy for enhanced variant detection. Methods such as Bayesian colocalization [33,34,35] evaluate whether cross trait associations share a common causal variant through posterior probability estimation, strengthening causal inference. Importantly, pleiotropic loci often implicate shared biological pathways. Integrating these genetic pleiotropic signals with functional genomic annotations can thus yield novel insights into disease pathophysiology and uncover potential therapeutic targets.

Epidemiological studies have established significant associations between AF and a range of cardiometabolic and behavior components [36,37,38,39,40,41,42,43,44]. In 2022, the American Heart Association (AHA) distilled the most pivotal of these into a framework for cardiovascular health, termed Life’s Essential 8 (LE8) [45], which comprises blood pressure (BP), blood lipids (LIP), blood glucose (GLU), obesity (OBS), smoking (SMK), physical activity (PA), diet (DIET), and sleep (SLP). LE8 provides a standardized, universally recognized, and comprehensive index of cardiovascular health that captures both behavioral and metabolic dimensions. Capitalizing on the underlying relationships between AF and LE8 components to identify pleiotropic loci may not only reveal susceptibility loci for AF undetectable by conventional GWAS but also advance our understanding of their biological functions and provide new pathophysiological insights into AF.

In this study, we integrated the largest GWAS summary statistics for AF and LE8 components that were available at the time of our analysis in individuals of European ancestry. First, we estimated genetic correlations between AF and each LE8 trait. Next, we identified pleiotropic variants and loci for each trait pair. Subsequently, we detected loci harboring shared causal variants across traits. Using these colocalized loci, we applied region-based methods and functional predictors to prioritize candidate genes. We then performed comprehensive functional annotations, including gene characterization, pathway and tissue expression enrichment, and drug target analysis, to elucidate the biological relevance of the prioritized genes. Finally, given that AF is a known precursor to serious cardiovascular and cerebrovascular events [36,46,47], we constructed a polygenic risk score (PRS) from the pleiotropic variants. This approach allowed us to explore the broader phenotypic relevance of our genetic findings by assessing the PRS associations not only with incident AF but also with a spectrum of related cardiovascular diseases (CVDs) in the UK Biobank population.

## 2. Materials and Methods

**Figure 1:**
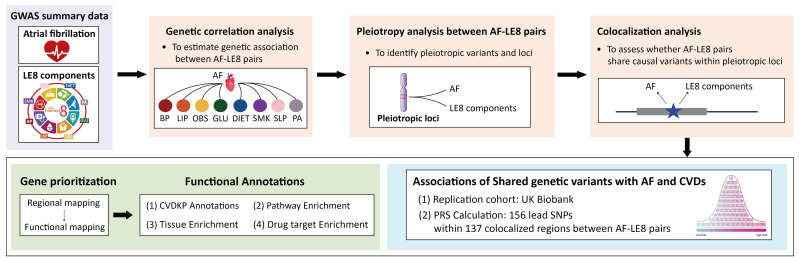
Flowchart of the study. The figure illustrates the overall workflow of the study, including data sources and analytical procedures. In the colocalization panel, the blue star denotes the causal variant, and the associated grey area represents the pleiotropic loci.

An overview of the study design and analytical workflow is presented in Figure 1.

### 2.1. GWAS Datasets

Traits corresponding to LE8 components were analyzed including BP (systolic BP, SBP; diastolic BP, DBP; pulse pressure, PP); LIP (triglycerides, TG; total cholesterol, TC; low-density lipoprotein cholesterol, LDLC; non-high-density lipoprotein cholesterol, NHDLC; high-density lipoprotein cholesterol, HDLC); OBS (body mass index, BMI; waist-to-hip ratio, WHR; waist-to-hip ratio adjusted for BMI, WHRadBMI); GLU (type 2 diabetes, T2D); DIET (healthy food consumption, DIEThf; overall healthy diet, DIETohd; the principal component-derived dietary pattern 1–5, DIETpc1-DIETpc5); SMK (smoking initiation status, SMKinit; age of smoking initiation, SMKage; cigarettes per Day, SMKcigday; smoking cessation status, SMKces); SLP (sleep duration, SLPdur; short sleep duration, SLPshort; long sleep duration, SLPlong); and PA (moderate-to-vigorous intensity physical activity during leisure time, Paint; Leisure screen time, PAlst; sedentary commuting, PAsedcm; and sedentary behavior at work, PAsedwk). Summary statistics for AF [48] and LE8 traits [49,50,51,52,53,54,55,56,57] were obtained from the largest published GWASs in individuals of European ancestry. Dataset details are provided in Table S1. All single-nucleotide polymorphisms (SNPs) were mapped to the GRCh38/hg38 reference genome, and the major histocompatibility complex region was excluded from analyses.

### 2.2. Genetic Correlation Analysis

Genetic correlations (r_g_) between AF and each LE8 trait were estimated using linkage disequilibrium score (LDSC) regression [58,59]. LD scores were computed using the 1000 Genomes Project Phase 3 European samples as the reference panel [60]. Significant genetic correlations are determined by Bonferroni correction as 0.05/30, resulting in a significance threshold of *p* < 1.67 × 10^−3^.

### 2.3. Pleiotropy Analysis

The condFDR approach [32] was applied to identify pleiotropic variants and to evaluate pleiotropic enrichment of AF, conditioning on LE8 traits. Variants with a condFDR value below 0.01 were considered significant. Independent genomic loci were defined using the functional mapping and annotation of genetic associations (FUMA) tool [61]. Loci were initially identified by grouping SNPs in linkage disequilibrium (r^2^ ≥ 0.6) with a significant lead SNP. Loci located within 250 kb were merged, and lead SNPs were determined using a clumping threshold of r^2^ < 0.1. These loci represent genomic regions associated with AF conditioning on specific LE8 traits.

### 2.4. Colocalization Analysis

Bayesian colocalization analysis was performed using the SuSiE algorithm implemented in the COLOC R package (version 5.2.3) to assess whether AF and LE8 traits share causal variants within the independent pleiotropic loci identified via condFDR [33]. Signed linkage disequilibrium matrices were generated using LDlinkR. Prior probabilities were set as *P*_1_ (1 × 10^−4^, SNP associated with trait 1 only), *P*_2_ (1 × 10^−4^, trait 2 only), and *P*_12_ (1 × 10^−5^, both traits). Posterior probabilities were calculated for each hypothesis, with hypothesis 4 (H_4_) indicating colocalization (i.e., shared causal variant/variants). A posterior probability for H4 (*PPH*_4_) exceeding 80% was used as the threshold for strong evidence of colocalization as recommended by the original methodology [33]. Given the extensive number of colocalized loci identified across the 30 AF-LE8 trait pairs, overlapping loci were consolidated into distinct colocalized regions containing all original colocalized lead SNPs to streamline the presentation of the results.

### 2.5. Gene Prioritization Analysis

A two-stage strategy incorporating five distinct lines of evidence was designed to prioritize candidate genes within colocalized loci. In the first stage, all genes located within colocalized loci were comprehensively identified using two region-based tools: (1) ANNOVAR (updated 5 December 2016) [62], which integrates positional information relative to genes, genomic regions, and existing databases; and (2) Ensembl Variant Effect Predictor (VEP [63], accessed 18 April 2025), which incorporates transcript-, protein-, and regulatory region-based annotations. In the second stage, candidate genes were further screened and prioritized by integrating the following predictors: (1) Variant-to-Gene (V2G) analysis (updated 19 March 2025) [64], leveraging gene expression, protein abundance, chromatin interaction and conformation data; (2) Polygenic Priority Score (PoPS, version 0.2) [65], which utilizes polygenic signals including single-cell RNA-seq data through similarity-based scoring; and (3) Summary-MultiXcan (SmultiXcan, version 0.6.1) [66], a transcriptome-wide association method for multi-tissue expression imputation. Within each locus, genes showing the highest V2G score, highest PoPS, or lowest SmultiXcan *p* value were considered candidate genes for AF. Genes simultaneously meeting all three criteria within a locus were designated as prioritized genes.

To determine whether the candidate genes identified in this study had been previously implicated in AF, three complementary approaches were employed to evaluate existing research evidence. First, the GWAS Catalog (up to 23 April 2025) [67] was queried to assess whether any of the candidate genes had been reported in prior AF GWAS. Second, Locus-to-Gene (L2G) scores [64] were utilized to systematically evaluate the likelihood of relationships between the candidate genes and known AF-associated loci. Finally, Human Genetic Evidence (HuGE) scores [68] were applied to quantify the cumulative genetic evidence supporting each candidate gene across all published AF genetic studies. Based on these three approaches, candidate genes absent from all three databases were classified as novel AF-associated genes.

### 2.6. Functional Annotation Analysis

To investigate the functional relevance of candidate genes in established cardiovascular and metabolic disease contexts, all candidate genes were queried using the Cardiovascular Disease Knowledge Portal (CVDKP; RRID:SCR_016536; https://cvd.hugeamp.org/ (accessed on 12 May 2025)). Documented gene functions and associated PubMed identifiers (PMIDs) were systematically extracted.

### 2.7. Pathway Enrichment Analysis

To investigate the biological mechanisms underlying the candidate genes, pathway enrichment analysis was performed using g:Profiler [69] (https://biit.cs.ut.ee/gprofiler (accessed on 24 April 2025)). Significantly enriched pathways were identified from Gene Ontology (GO) databases using a cumulative hypergeometric test with multiple testing correction via the g:SCS method [70], applying a significance threshold of 0.05. To account for correlations among GO terms, significant terms were first reorganized based on their ontological relationships and clustered into connected components representing similar biological contexts (designated as “highlight” pathways), thereby facilitating result interpretation.

### 2.8. Tissue Enrichment Analysis

To assess tissue-specific expression patterns of candidate genes identified from LE8-AF trait pairs, tissue enrichment analysis was performed using the GENE2FUNC module implemented in the FUMA platform (https://fuma.ctglab.nl (accessed on 10 June 2025)) [61].

### 2.9. Drug Target Analysis

To evaluate associations between candidate genes and approved drugs and to explore the potential drug repositioning opportunities for AF treatment, functional enrichment analysis of drug targets was performed using GREP (genome for REPositioning drugs, version 1.0.0) [71]. A series of Fisher’s exact tests was conducted to assess whether the candidate gene set was significantly enriched for genes targeted by drugs within specific Anatomical Therapeutic Chemical (ATC) classification categories. To account for multiple hypothesis testing across 90 detailed ATC classes, statistical significance was determined by Bonferroni correction as 0.05/90, resulting in a significance threshold of *p* < 5.56 × 10^−4^.

### 2.10. Association Analysis of Shared Genetic Variants with AF and CVDs

To explore the clinical relevance of AF related loci identified by conditioning on LE8 components, two PRSs were constructed and evaluated for their associations with incident AF in the UK Biobank. The AF-LE8-PRS was constructed using lead SNPs from all colocalized loci identified across AF-LE8 trait pairs. Given the predominance of blood pressure (BP)-related signals among AF-LE8 associations, an AF-BP-PRS was additionally derived using lead SNPs specifically from colocalized loci identified in the three BP-AF trait pairs. PRS calculations were performed using the PRS pipeline [72] (https://2cjenn.github.io/PRS_Pipeline (updated 29 March 2022)). These scores were subsequently evaluated for associations with incident AF and a spectrum of CVDs using Cox proportional hazards models for continuous scores, adjusted for the top ten genetic principal components to account for population stratification. CVD outcomes included overall CVD and the following 14 subtypes: rheumatic heart diseases (RHD), hypertensive diseases (HD), ischemic heart diseases (IHD), cor pulmonale (CP), pericardial diseases (PD), valvular heart diseases (VHD), myocardial diseases (MD), arrhythmia (AR), heart failure (HF), other heart diseases (OHD), cerebrovascular disease (CED), diseases of arteries, arterioles and capillaries (AAD), diseases of veins, lymphatic vessels and lymph nodes (VLD), and other and unspecified disorders of the circulatory system (OCD). Hazard ratios (HRs) were estimated and statistical significance was determined by Bonferroni correction across AF, overall CVDs and 14 subtypes above as 0.05/16, resulting in the significance threshold of *p* < 3.13 × 10^−3^.

## 3. Results

### 3.1. Genetic Correlations Between AF and LE8 Components

**Figure 2:**
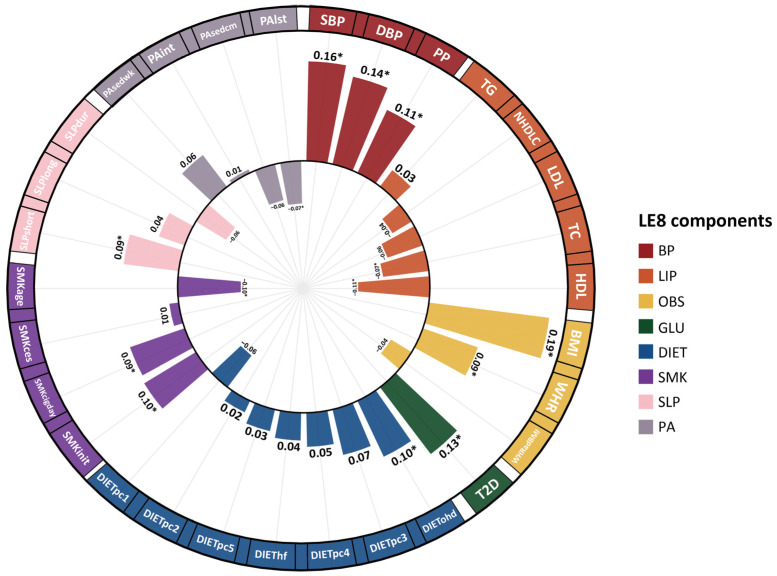
Genetic Correlations between AF and LE8 components. The plot displays the genetic correlation (r_g_) between AF and each LE8 trait from linkage disequilibrium score (LDSC) regression. Thirty traits are arranged on the outer ring and color-coded by their LE8 component in the legend. Bar length represents the magnitude of r_g_, with direction outward and inward indicating positive and negative correlations, respectively. The r_g_ values are provided at bar tips. Statistically significant genetic correlations are determined by Bonferroni correction as 0.05/30, resulting in a significance threshold of *p* < 1.67 × 10^−3^, denoted by the asterisks (*) in the bar tips.

Across the LE8 components, r_g_ estimates for AF ranged from −0.11 to 0.19, with statistically significant genetic correlations identified between AF and at least one measured trait within each component. Among all statistically significant r_g_ values, cardiometabolic components exhibited generally stronger genetic correlations with AF compared to behavioral components. The most substantial positive correlations were observed for BMI (r_g_ = 0.19), SBP (r_g_ = 0.16), DBP (r_g_ = 0.14), T2D (r_g_ = 0.13), and PP (r_g_ = 0.11), while HDLC showed the strongest negative correlation (r_g_ = −0.11). BP was the only component where all measured traits demonstrated significant genetic correlations with AF (Figure 2).

### 3.2. Pleiotropic Genomic Loci Between AF and LE8 Components

Analysis of shared genetic architecture between AF and LE8 components revealed substantial pleiotropy across all trait pairs (Figures S1–S30, Table S2). In total, 26,766 pleiotropic variants distributed across 970 independent genomic loci were identified. These loci were widely distributed throughout the genome, with notable clustering observed on chromosomes 1, 2, 5, 6, 7, 12, 17 and 22 (Figure 3A). Among individual LE8 components, BP showed the highest number of pleiotropic variants (24,709) and loci (639) shared with AF, followed by LIP (22,375 variants and 550 loci) and OBS (20,946 variants and 489 loci) (Figure 3A, Table S2). These findings were consistent with fold enrichment analysis, which demonstrated significant enrichment of AF-associated loci for BP-related traits, with enrichment values reaching approximately 10-fold (Figure 3B).

### 3.3. Colocalized Loci with Shared Causal Variants Between AF and LE8 Components

**Figure 3:**
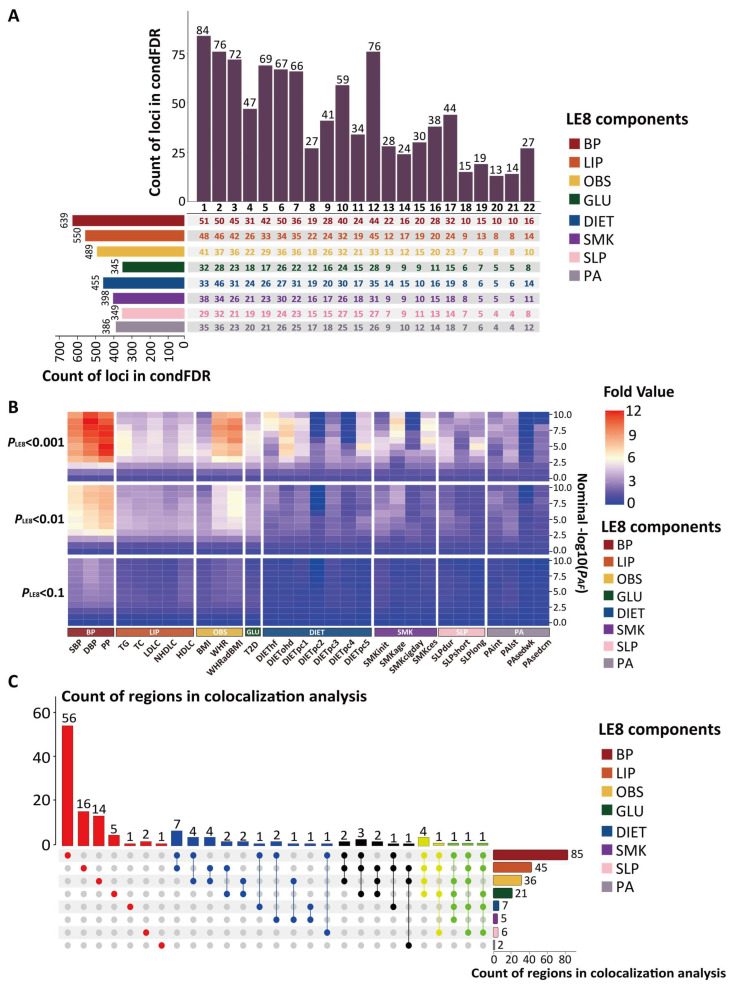
Pleiotropy and colocalized loci between AF and LE8 components. (**A**) The distribution of pleiotropic loci across chromosomes and AF-LE8 trait pairs identified by conditional false discovery rate (condFDR) approach. The horizontal bar plot displays the count of loci across chromosomes 1–22 labeled in the x-axis. The exact numbers of total counts within each chromosome are labeled above each vertical bar. The vertical bar plot represents the count of loci in each AF-LE8 pair with different colors as defined in the legend. (**B**) The fold enrichment of pleiotropy between AF and LE8 components from condFDR. The heatmap illustrates the fold enrichment, calculated as the ratio of the −log10(*P_AF_*) cumulative distribution of SNPs conditional on *P_LE_*_8_ thresholds <0.1, <0.01, and <0.001 for each LE8 component to the cumulative distribution of all SNPs for that LE8 component, with colors ranging from blue (lower enrichment) to red (higher enrichment). (**C**) The distribution of 137 distinct colocalized regions containing colocalized loci across AF-LE8 trait pairs identified by colocalization analysis. The horizontal bar plot illustrates the count of loci that colocalize with AF and at least one LE8 components. The matrix of colored circles connected by lines indicates specific AF-LE8 trait pairs corresponding to the specific bar above. The vertical bar chart on the right shows the total counts of colocalized loci for each AF-LE8 pairs, with different bar colors indicating categories based on the number of LE8 components: red bars represent colocalization with only one LE8 component, blue with two, black with three, yellow with four, and green with five.

Among 970 pleiotropic loci, 179 were identified as colocalized loci that demonstrated evidence of shared causal variants (*PPH*_4_ > 0.8) between AF and at least one LE8 component. These colocalized loci were encompassing 137 distinct colocalized regions and 156 lead SNPs distributed genome-wide (Figure 3C, Table 1). The highest number of these colocalized regions was identified for AF-BP trait pairs (85), followed by AF-LIP (45), AF-OBS (36), AF-GLU (21), AF-DIET (7), AF-SLP (6), AF-SMK (5), and AF-PA (2) trait pairs (Figure 3C). Of these colocalized regions, 95 (69%) were shared exclusively between AF and a single LE8 component, while the remainder exhibited shared genetic influence across multiple components: 25 regions with two components, 9 with three, 5 with four, and 3 regions shared with five LE8 components. BP contributed the largest number of colocalized loci in both the AF-exclusive (56 regions) and multi-component (29 regions) categories. Three regions showed particularly extensive pleiotropy, being shared between AF and five different LE8 components: a region on chromosome 3 (27230230-141154542; lead SNPs rs6763931 and rs73046176) shared with BP, BMI, T2D, DIET, and SMK; a region on chromosome 6 (100600097-100629461; lead SNP rs17185536) shared with BP, LIP, BMI, DIET and SLP; and a region on chromosome 16 (53797908-53848561; lead SNP rs9941349) shared with BP, LIP, T2D, DIET and SLP.

### 3.4. Gene Prioritization

**Figure 4:**
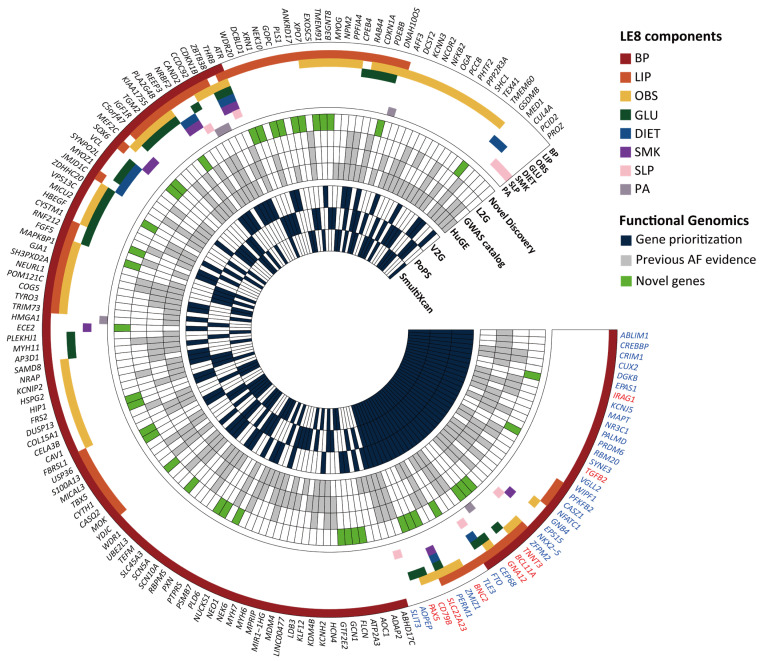
Prioritization of candidate genes from colocalized loci between AF and LE8 components. This figure illustrates the evidence of gene prioritization and the confirmation of novel AF-associated genes. The innermost circle displays the functional genomics evidence of candidate genes and fills the cells in dark blue if they rank highest in V2G score or PoPS score, or have the smallest SmultiXCAN *p* value within their locus. Prioritized genes meeting simultaneously the three criteria above are highlighted in blue of the font. The second circle presents the previous AF evidence for the confirmation of novel AF-associated genes. The cells in the second circle in light gray means that the genes were supported in the evidence of HuGE, GWAS catalog, or L2G (see Section 2 for details). And novel AF-associated genes lacking all three are filled in light green in the cells of “Novel discovery”. Genes designated as both prioritized and novel AF genes are labeled in red of the font, while other candidate genes are in black. The third circle illustrates the specific LE8 components of colocalized loci harboring the specific candidate gene.

A two-stage gene-mapping strategy identified 164 candidate genes, including 38 prioritized genes supported by three lines of evidence (V2G, PoPS, and SmultiXcan). Cross-referencing with the GWAS Catalog, L2G, and HuGE score databases revealed 40 novel AF-associated genes, 9 of which were prioritized genes: *CD79B*, *IRAG1*, *SLC22A23*, *TNNT3*, *PAX5*, *BCL11A*, *TGFB2*, *BNC2*, and *GNA12*. Most candidate (118/164) and prioritized (29/38) genes were mapped to AF-BP colocalized loci (Figure 4, Table S3).

### 3.5. Functional Annotation and Enrichment

#### 3.5.1. Functional Annotation

Functional annotation through the CVDKP indicated that the majority of the 164 candidate genes have established associations with cardiometabolic disorders, neurodegenerative diseases, and cancers (Table S3). These genes participate in diverse biological processes, including cardiac contractility, cellular signaling, transcriptional regulation, cytoskeletal formation, and cell cycle regulation. Among the 40 novel AF-associated genes, functional enrichment highlighted roles in transcription factor activity and signal transduction. Nine of these novel genes were prioritized, most with documented relevance to cardiovascular pathology: *CD79B* has been linked to coronary artery disease; *IRAG1* encodes a substrate for cGMP-dependent kinase 1 (PKG1) and modulates cGMP-PKG signaling in blood pressure regulation and pulmonary hypertension; *SLC22A23* is implicated in drug-induced QT interval prolongation; *TNNT3*, encoding fast skeletal troponin T, has been associated with atrial septal defect; *PAX5* encodes a pairing box family transcription factor and participates in ferroptosis in cardiomyocytes within injury-related cardiac regions (e.g., border, ischemic, and fibrotic zones); *BCL11A*, encoding a C2H2-type zinc finger protein, is linked to the transition from AF to sinus rhythm during recovery; *TGFB2*, encodes a TGF-β superfamily ligand and serves as a diagnostic locus for Loeys–Dietz syndrome, which features aortic and peripheral aneurysms; and *GNA12* regulates myocardial mitophagy and confers ischemic protection via RHOA signaling (Table S3).

#### 3.5.2. Pathway Enrichment

**Figure 5:**
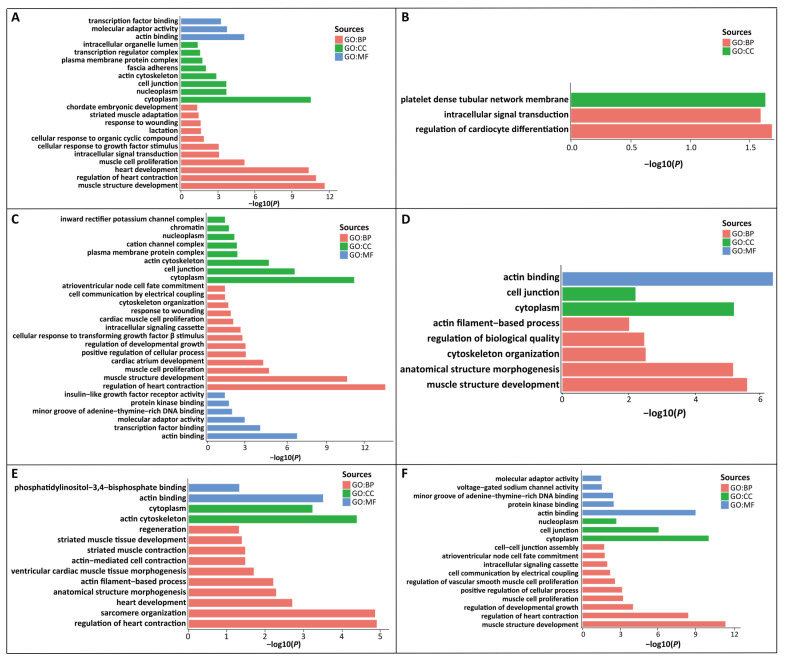
Pathway enrichment of candidate genes. (**A**) Enrichment results for all candidate genes. (**B**) Enrichment results for novel AF candidate genes. (**C**) Enrichment results for candidate genes from AF-BP colocalized loci. (**D**) Enrichment results for candidate genes from AF-SBP colocalized loci. (**E**) Enrichment results for candidate genes from AF-DBP colocalized loci. (**F**) Enrichment results for candidate genes from AF-PP colocalized loci. In each panel, bars represent the highlighted pathways after multiple testing correction (*p* < 0.05) via the g:SCS method and clustering by g:profiler.

Pathway enrichment analysis identified 195 significantly enriched pathways (Table S4). Twenty-two representative Gene Ontology terms were highlighted based on genetic evidence, gene counts, and statistical significance (Figure 5A). The five most significantly highlighted pathways included muscle structure development, regulation of heart contraction, heart development, cytoplasm, and muscle cell proliferation (Figure 5A). The 40 novel AF-associated genes were significantly enriched in three highlighted pathways: regulation of cardiomyocyte differentiation, intracellular signal transduction, and the platelet dense tubular network membrane pathway (Figure 5B, Table S5). Given that most candidate genes mapped to AF-BP colocalized loci, pathway enrichment analysis was specifically performed on the 118 candidate genes from AF-BP colocalized loci (Figure 5C, Table S6). The resulting pathways were broadly consistent with those from the full candidate gene set, showing enrichment for biological processes related to structural development and ion channel activity/electrical signaling. Pathways associated with structural development, such as muscle structure development, actin binding, and anatomical morphogenesis, were predominantly enriched by genes colocalized with SBP or DBP (Figure 5D,E, Tables S7 and S8). In contrast, pathways related to PP not only encompassed structural development, such as muscle structure development and actin binding, but also extended to ion channel activity and electrical signaling, such as voltage-gated sodium channel activity and electrical synapse-mediated communication (Figure 5F, Table S9).

#### 3.5.3. Tissue Specificity

**Figure 6:**
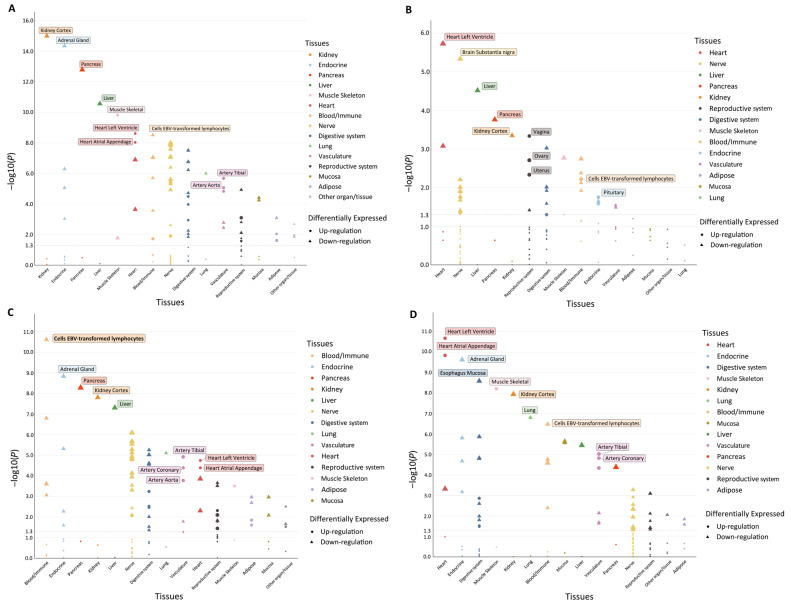
Tissue Enrichment of candidate genes. (**A**) Enrichment results for all candidate genes. (**B**) Enrichment results for novel AF-associated candidate genes. (**C**) Enrichment results for candidate genes identified from AF-SBP and/or AF-DBP colocalized loci. (**D**) Enrichment results for candidate genes identified from AF-PP colocalized loci. In each panel, circles represent upregulation of genes in the tissues on the x-axis, while triangles represent downregulation. Symbol size is proportional to the number of enriched genes in each tissue, with larger symbols indicating a greater number of genes, and color denotes the tissue category in the legend. The dashed line indicates the significance threshold of *p* < 0.05. Data points above this dashed line represent statistically significant differences in the tissue. The top five genes in upregulated tissues and the top five genes in downregulated tissues are labeled, respectively.

Candidate genes showed elevated expression in skeletal muscle, cardiac tissues (including left ventricle and atrial appendage), and arterial tissues (e.g., tibial artery and aorta), with downregulation observed in the kidney cortex, adrenal gland, pancreas, liver, blood/immune, nervous, lung, and digestive system (Figure 6A). The 40 novel AF-associated genes exhibited increased expression in vaginal tissue but reduced expression in cardiac tissues (left ventricle and atrial appendage), substantia nigra, pancreas, liver, kidney cortex, and digestive system (Figure 6B). Genes from AF-BP colocalized loci displayed tissue-enrichment profiles similar to the full candidate set, showing elevated expression in left ventricle, atrial appendage, tibial artery, skeletal muscle, and coronary artery. The tissue enrichment profiles of genes from AF-SBP/DBP and AF-PP colocalized loci were largely similar across tissues, except for a significantly elevated expression of AF-PP genes in cardiac tissue (Figure 6C,D).

#### 3.5.4. Drug Target Enrichment

Candidate genes showed significant enrichment for Otologicals (ATC category S02, *p* = 2.37 × 10^−4^), as well as suggestive enrichment for three additional drug categories, including Vasoprotectives (ATC category C05, *p* = 3.12 × 10^−3^), Antipruritics (ATC category D04, *p* = 4.55 × 10^−3^), and Throat preparations (ATC category R02, *p* = 2.74 × 10^−3^). Notably, *NR3C1*, *SCN5A* and *SCN10A* were associated with both otological and vasoprotective agents, with *NR3C1* enriched for glucocorticoids (e.g., hydrocortisone), and both *SCN5A* and *SCN10A* enriched for local anesthetics (e.g., lidocaine) (Table 2).

### 3.6. Associations of Shared Genetic Variants with AF and CVDs Risk

**Figure 7:**
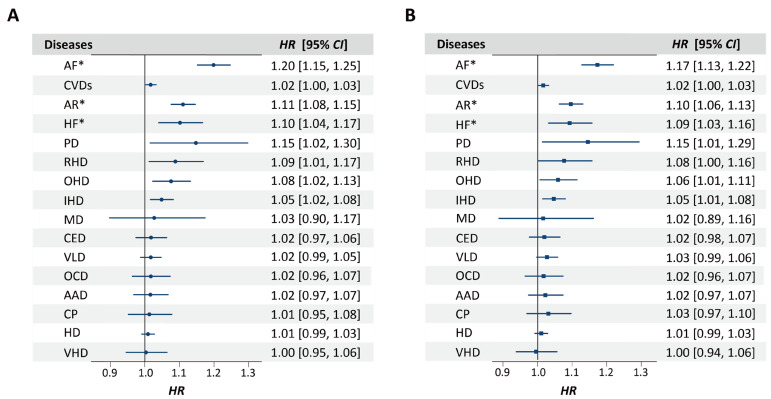
Associations of shared genetic variants with AF and CVDs risk in the UK Biobank. (**A**) Associations of AF-LE8-PRS with AF and CVDs. (**B**) Associations of AF-BP-PRS with AF and CVDs. In each panel, *HR* and 95% *CI* were used to describe the risk of each disease, with the forest plot and values presented. The asterisks (*) next to the disease name indicate statistical significance at the Bonferroni-corrected level (*p* < 3.13 × 10^−3^).

PRSs for AF were constructed based on colocalized loci from AF-LE8 (AF-LE8-PRS) and AF-BP (AF-BP-PRS) trait pairs and evaluated for association with AF incidence in the UK Biobank. Each SD increase in the AF-LE8-PRS and AF-BP-PRS was associated with a higher AF risk, with an increase of 19.9% (*HR* = 1.20, 95% *CI*: 1.15–1.25, *p* < 2.00 × 10^−16^) and 17.3% (*HR* = 1.17, 95% *CI*: 1.13–1.22, *p* = 1.21 × 10^−15^) respectively. No significant difference was observed between the two PRS models as measured by C-index for AF (*p* = 0.143). Both continuous PRSs were also significantly associated with AR and HF after Bonferroni correction (Figure 7).

## 4. Discussion

Leveraging a pleiotropy-informed framework conditioned on LE8 traits, we conducted an in-depth investigation into the genetic susceptibility of AF. Our analysis yielded several significant findings. First, we established a substantial shared genetic basis between AF and all LE8 components, which facilitated the identification of a considerable number of AF-associated loci. Second, we identified 179 colocalized loci mapping to 137 distinct colocalized regions harboring shared causal variants for one or more AF-LE8 trait pairs. These loci collectively pointed to 164 AF-susceptibility genes, 40 of which were novel, potentially involving the pathways of heart development, cardiac contraction regulation, muscle cell proliferation, muscle structure development and cytoplasmic processes. Third, among all LE8 components, BP emerged as the LE8 component exhibiting the most prominent genetic overlap with AF. This is supported by the strongest genetic correlation, the degree of pleiotropic enrichment, and the largest number of shared genetic loci with AF. Finally, the colocalized loci identified in this study demonstrate notable clinical relevance, exhibiting associations with both AF and several subtypes of CVDs including AR and HF, as well as enrichments for drug targets related to otological agents.

We observed statistically significant genetic correlations between AF and all LE8 components, consistent with known phenotypic and genetic links [1,73,74]. Some r_g_ magni-tudes for AF-LE8 trait pairs were modest, this may be attribute to the inherent complexity of the traits’ genetic architecture, the extent of variants captured by current GWASs, and the potentially modest genetic links within the specific trait pairs. Nevertheless, cardiometabolic components showed stronger genetic correlations with AF than behavioral ones, suggesting a closer etiological link to AF pathogenesis. Among cardiometabolic traits, BP metrics (SBP, DBP, and PP) exhibited the strongest associations, suggesting an important role of hemodynamic factors in AF development. For obesity related traits, BMI, reflecting overall adiposity, showed a stronger association with AF than WHR, a measure of central adiposity, a pattern consistent with prior reports [73]. Furthermore, lipid traits consistently showed inverse genetic correlations with AF, including an unexpected negative association with TC. This pattern aligns with recent reports [73] and is consistent with the “cholesterol paradox” [75]. Potential explanations for this inverse relationship include methodological factors (e.g., Neyman’s bias [76,77,78], residual confounding [79]), or other complex biological mechanisms.

Our pleiotropy-based approach identified 164 AF susceptibility genes, including both established and novel candidate genes. Most of these genes were mapped from colocalized loci shared between AF and cardiometabolic risk factors, consistent with our genetic correlation findings. A subset of these genes, including *KCNN3* [19,26], *HCN4* [12,26], *SYNPO2L* [12,26], *MYOZ1* [12], *SCN5A* [48,80], *KCNH2* [23,48], *TBX5* [20,26,48,81,82], *NKX2-5* [48,80,81,82], *MYH7* [20,48,82], *SCN10A* [80], and *GJA1* [26,81], had been previously implicated in AF through GWAS or candidate gene studies [12,19,20,23,26,48,80,81,82]. These genes converge on three major biological pathways relevant to AF pathogenesis, including cardiac electrophysiology, structural remodeling, and transcriptional regulation. Among these candidate genes, 40 represented novel AF associations. Of these novel genes, 25 showed pleiotropy with BP, 22 with blood lipids, 15 with obesity metrics, seven with T2D, four with smoking, and two with exercise- and diet-related traits, respectively. The pleiotropic pattern of these newly identified genes mirrored that of the full candidate set, with most being mapped from loci colocalized with cardiometabolic traits.

Among all LE8 components, BP emerges as the factor most strongly associated with AF, a conclusion corroborated by multiple lines of evidence from both our observed genetic overlap and the comparable associations of AF-BP-PRS and AF-LE8-PRS with AF in the present study and prior global burden studies [3,83]. Mendelian randomization studies have further established the causal effects of genetically elevated BP levels on AF risk [84]. However, the relationship between elevated BP and AF is likely bidirectional. AF itself may elevate BP variability and sustain elevated PP even after rhythm restoration accelerating arterial stiffness and increasing the risk of adverse CVD outcomes, an interplay that has been acknowledged in recent clinical consensus recommendations [85]. This pronounced genetic overlap between AF and BP, relative to other LE8 components, may be partially attributed to the substantial statistical power of the large-scale GWAS summary statistics used in our pleiotropic analyses, a point that will be addressed in detail in the limitations. Additionally, the subtle differences observed in the pathway enrichment of genes mapped to AF-SBP/DBP (predominantly structural development) and AF-PP (extending to ion channel activity) colocalized loci suggest distinct underlying mechanisms. SBP and DBP largely reflect peripheral vascular resistance, whereas PP is more directly related to pulsatile stress from intrinsic cardiac contraction [86,87,88,89,90]. Epidemiological evidence suggests that SBP and DBP are stronger CVDs risk markers in younger individuals, while PP gains predictive value in middle-aged and older adults [89]. Collectively, these results suggest that AF risk management may benefit from BP strategies tailored to specific pressure components and age groups.

AF pathogenesis involves highly complex mechanisms. While animal and human studies indicate that atrial histological alterations (fibroblast proliferation, extracellular matrix changes, myocyte hypertrophy, etc.) can initiate AF [91] by shortening the left atrial refractory period and promoting re-entry circuits, this classical framework may be inadequate for fully deciphering the disease. By querying the GWAS Catalog, we revealed widespread pleiotropy among our identified AF-associated genes, extending far beyond cardiovascular traits. For instance, *MEF2C* and *BNC2* were significantly associated with hundreds of diverse traits. These genes showed pleiotropic effects across multiple physiological domains, including systemic metabolic regulation (renal and liver function indices), hematologic parameters, skeletal integrity (bone mineral density), pulmonary function, gut microbiome features, brain MRI phenotypes, neurological and psychiatric disorders, and inflammation-related conditions, as well as sex and sex hormone-related traits. This extensive pleiotropy represents both a challenge and an opportunity. While it complicates mechanistic dissection, it profoundly reflects the systemic nature of human physiology. Although AF primarily manifests in the heart, its genetic architecture implies deep interconnectivity with other organ systems. Established concepts such as the heart–brain axis [92], gut–heart axis [93], and cardio–renal axis [94,95,96] may represent macroscopic manifestations of this underlying genetic pleiotropy. This extensive genetic pleiotropy suggests that the prevention and treatment of AF may require shifting focus beyond the heart itself toward systemic interventions. Consequently, we must not only deeply dissect these cross-system pathogenic pathways but also leverage this understanding to identify opportunities for the “repurposing of old drugs.” Furthermore, these novel identified associated genes expand the range of potential targets available for future gene therapies, such as *TGFB2*. Prioritized as a novel AF-associated gene by our pleiotropic framework, *TGFB2* has recently been highlighted as a gene therapy target for AF by mitigating atrial fibrosis and improving cardiac conduction, underscoring the profound value of integrating correlated traits via such pleiotropic approaches to yield clinical implications for AF [97].

Our pleiotropy-informed strategy demonstrated high research efficiency by identifying novel AF-associated genes and recovering rare variant loci that typically require resource-intensive sequencing. During the preparation of this manuscript, three recent AF genetics studies utilizing substantially larger sample sizes (>120,000 AF cases) or diverse populations were published [28,98,99]. Comparative analysis revealed that 16 of our 40 novel genes were independently validated in those studies, confirming the robustness of our pleiotropy-based approach. Notably, we successfully recovered *SCN10A* [100] and *GJA1* [101], both initially linked to AF through rare-variant studies. The identification of these critical electrophysiological genes, which typically require large sample sizes and deep sequencing in rare-variant analyses, underscores the efficacy and reliability of our strategy in uncovering high-confidence AF susceptibility loci.

Our study has several limitations. First, although we identified multiple AF susceptibility genes, including novel candidates, our findings were derived exclusively from associations in the population level. Second, despite selecting the largest available European-ancestry GWAS datasets for each trait at the time of analysis to maximize statistical power and mitigate sample size discrepancies as much as possible, the pleiotropy analysis was constrained by the limited set of risk factors and the uneven sample sizes across the GWAS summary statistics used. Notably, the finding that BP harbored the most pleiotropic loci with AF may be largely driven by its markedly larger sample size. Considering the multifactorial nature of AF pathogenesis, subsequent studies should incorporate a broader and more balanced array of clinical and molecular traits to fully elucidate the underlying biology. Third, all analyses were restricted to European ancestry, which may limit the generalizability and translational relevance of our findings to other ethnic groups. Fourth, there is partial sample overlap between the AF GWAS dataset used for pleiotropy analysis and the UK Biobank cohort used for PRS evaluation, which may potentially inflate the PRS–disease association estimates.

## 5. Conclusions

In summary, our study identified multiple novel AF susceptibility genes, substantially expanding the genetic evidence for AF. The observed extensive pleiotropy provides a novel paradigm for understanding the genetic architecture of AF. Our approach underscores the broad translational utility of pleiotropy-informed strategies in dissecting the complex etiology of cardiovascular diseases.

## Figures and Tables

**Table 1:** Colocalized regions containing colocalized loci between AF and LE8 components.

	Colocalized Regions	Lead SNPs	Candidate Genes	Range of *PPH*_4_	Average of *PPH*_4_
1	1:908823:918617	rs7417106	*PERM1*	0.88–0.98	0.93
2	1:10758063:10802977	rs880315	*CASZ1*	0.99–1.00	1.00
3	1:22203808:22345647	rs7529220	*CELA3B*, *HSPG2*	0.87–0.99	0.93
4	1:50885622:87965485	rs12141405, rs17106497	*EPS15*	0.90–0.98	0.94
5	1:100116253:100164547	rs1933723	*PALMD*	0.94–0.94	0.94
6	1:116214111:153668678	rs4073778, rs9330300	*CASQ2*, *S100A13*	0.82–0.96	0.89
7	1:154394297:156063880	rs1218582	*DCST2*, *KCNN3*, *SHC1*	0.85–0.85	0.85
8	1:203007612:203108508	rs3737883	*MYOG*, *PPFIA4*	0.98–1.00	0.99
9	1:205641826:205744138	rs4951261	*MYOG*, *PPFIA4*	0.99–0.99	0.99
10	1:207193177:207251518	rs1044145	*PFKFB2*	0.87–0.97	0.91
11	1:204449952:204582730	rs2926536	*NUCKS1*, *SLC45A3*, *MDM4*	0.89–0.89	0.89
12	1:218523650:218701451	rs12078363	*TGFB2*	0.96–0.97	0.96
13	2:17816326:17991974	rs765396		0.83–0.83	0.83
14	2:36615195:36678210	rs17018699	*CRIM1*	0.87–0.87	0.87
15	2:65235333:145852801	rs11679718, rs13010262, rs2723064	*CEP68*, *TEX41*	0.83–1.00	0.93
16	2:46199356:46548064	rs11689011	*EPAS1*	0.88–0.98	0.93
17	2:60603950:60783572	rs2244030	*BCL11A*	0.82–0.99	0.94
18	2:100208830:100478648	rs12620982	*CEP68*, *AFF3*	0.89–0.89	0.89
19	2:161369556:161566840	rs13010247		0.98–0.98	0.98
20	2:175200127:175570413	rs7574892	*WIPF1*	0.99–1.00	0.99
21	2:208339501:208365979	rs10186845		0.90–0.90	0.90
22	3:12611269:72511359	rs11128268, rs73041705, rs7650482	*CAND2*, *THRB*	0.89–1.00	0.94
23	3:27230230:141154542	rs6763931, rs73046176	*THRB*, *ZBTB38*	0.80–0.96	0.93
24	3:14837473:14953936	rs1077534	*ZBTB38*	0.95–0.95	0.95
25	3:142002869:142409393	rs147812234	*NEK10*, *PLS1*, *ATR*, *XRN1*	0.84–0.89	0.87
26	3:179027192:179173969	rs7612445	*GNB4*	0.95–1.00	0.97
27	3:38579207:38826179	rs6801957	*SCN10A*, *SCN5A*	0.98–0.98	0.98
28	3:135621417:136712852	rs1278493	*PCCB*, *PPP2R3A*	0.92–0.92	0.92
29	3:133761861:133803719	rs79241561		0.97–0.97	0.97
30	3:185541213:185548683	rs720389		0.97–0.97	0.97
31	3:183992775:184003194	rs4912532	*GNB4*, *ECE2*	0.96–0.96	0.96
32	3:183992775:184006097	rs843341	*ECE2*	0.93–0.93	0.93
33	4:1036545:81207963	rs12640611, rs1458038, rs3816474	*RNF212*, *FGF5*, *WDR1*	0.83–0.99	0.91
34	4:73860920:74325956	rs56382488	*FGF5*, *ANKRD17*	0.82–0.82	0.82
35	5:87986239:88206889	rs412458	*MEF2C*	0.81–0.99	0.92
36	5:168347056:168426915	rs77328981	*SLIT3*	0.89–0.89	0.89
37	5:172474355:172690831	rs6891790	*NKX2-5*	0.97–0.99	0.98
38	5:76548799:132470830	rs10070672, rs1096752	*PDE8B*	0.91–0.97	0.95
39	5:32775047:32832474	rs2193950		0.97–0.97	0.97
40	5:139515394:173556628	rs17118812, rs56180201	*CYSTM1*, *HBEGF*, *C5orf47*, *CPEB4*	0.82–0.90	0.87
41	5:122442627:122596593	rs74661587	*PRDM6*	0.86–0.99	0.92
42	5:142494165:142978777	rs6580277	*NR3C1*	0.98–1.00	0.99
43	6:3446263:3468878	rs9503598	*SLC22A23*	0.85–0.94	0.93
44	6:19374744:19381743	rs9368060		0.97–0.97	0.97
45	6:100600097:100629461	rs17185536		0.98–1.00	0.99
46	6:31321519:36659932	rs3176326, rs3997995	*RAB44*, *CDKN1A*	0.92–0.99	0.96
47	6:120112137:120142181	rs7773530		0.85–0.85	0.85
48	6:117859112:117989912	rs11153672	*DCBLD1*, *GOPC*	0.90–0.90	0.90
49	6:140263827:140299541	rs596878		0.99–0.99	0.99
50	6:34153882:34397628	rs9469745	*HMGA1*	0.99–0.99	0.99
51	6:121323385:122940296	rs72966339	*GJA1*	0.85–0.93	0.89
52	6:117107704:117572603	rs11153653	*VGLL2*	0.95–0.95	0.95
53	6:135069013:135128858	rs4896104		0.99–1.00	1.00
54	7:2750193:2912928	rs1182178		0.88–0.91	0.90
55	7:75038408:75176196	rs62477684	*GNA12*, *HIP1*	0.92–0.97	0.95
56	7:77305841:77598538	rs848462	*PHTF2*, *TMEM60*	0.81–0.81	0.81
57	7:113053088:113228710	rs4546597	*GNA12*	0.87–0.87	0.87
58	7:106666157:150703915	rs1899689, rs7789585, rs9791733	*COG5*, *KCNH2*, *AOC1*	0.82–0.98	0.88
59	7:14353038:14386532	rs12154315	*DGKB*	0.92–0.92	0.92
60	7:26853846:27314815	rs10262140		0.81–0.83	0.82
61	7:115554257:116319214	rs55912035		0.99–1.00	1.00
62	8:21707357:116403541	rs188974793, rs6998692	*TRIM73*, *POM121C*, *XPO7*, *NPM2*	0.92–0.96	0.94
63	8:105885409:106026758	rs34007487	*ZFPM2*	0.96–0.98	0.97
64	8:30271637:30477733	rs6982511	*GTF2E2*, *RBPMS*	0.85–0.85	0.85
65	9:36997051:37003075	rs7020413	*PAX5*	0.85–0.91	0.88
66	9:33785245:34071144	rs10758239		0.82–0.85	0.84
67	9:96912177:98132992	rs7864171	*AOPEP*	0.82–0.82	0.82
68	9:101738188:101784121	rs1413299	*CAV1*, *COL15A1*	0.93–0.95	0.94
69	9:16713666:16906384	rs1411430	*BNC2*	0.98–0.98	0.98
70	9:20190349:20239894	rs7036564		0.92–0.92	0.92
71	9:126999153:127184067	rs10986332	*NEK6*, *PSMB7*	0.85–0.85	0.85
72	10:64579460:65400352	rs12245149	*NRBF2*, *JMJD1C*, *REEP3*	0.86–0.94	0.89
73	10:76839355:76998092	rs2002023	*DUSP13*, *SAMD8*	0.96–0.96	0.96
74	10:80776636:80898969	rs1769758	*ZMIZ1*	0.98–0.99	0.99
75	10:103047393:104197327	rs11191116	*NFKB2*, *OGA*, *KCNIP2*	0.86–0.86	0.86
76	10:104487382:105720351	rs10883886	*SH3PXD2A*, *NEURL1*	0.99–1.00	1.00
77	10:74819021:75695919	rs41280404	*MYOZ1*, *SYNPO2L*, *VCL*	0.94–0.98	0.96
78	10:103047393:116464387	rs11191804, rs12571587, rs2208322	*SH3PXD2A*, *NEURL1*, *ABLIM1*	0.92–1.00	0.98
79	10:88444220:88455242	rs3802662	*LDB3*	0.98–0.98	0.98
80	10:102912264:105720351	rs60848348	*KCNIP2*, *NEURL1*	0.99–1.00	1.00
81	10:112491620:112576695	rs10749053	*RBM20*	0.97–0.97	0.97
82	10:115390091:115511203	rs3121483	*FRS2*, *NRAP*	0.90–0.90	0.90
83	11:16231667:16373664	rs991612	*SOX6*	0.81–0.84	0.83
84	11:1874892:1949470	rs4980386	*TNNT3*	0.85–0.98	0.90
85	11:18700010:18711901	rs11606447		0.97–0.99	0.98
86	11:10654911:10660067	rs11042901	*IRAG1*	0.80–0.80	0.80
87	11:65255589:65346178	rs2510078		0.83–0.83	0.83
88	11:128764180:129158679	rs76097649	*KCNJ5*	0.97–0.97	0.97
89	12:12848551:12893723	rs10845620		0.99–1.00	1.00
90	12:124399155:124496316	rs11831913	*CDKN1B*, *DNAH10OS*, *CCDC92,*	0.81–0.88	0.85
91	12:21503467:21747633	rs4762849		0.99–1.00	1.00
92	12:66383320:66389968	rs10400419		0.98–0.99	0.99
93	12:69853934:70098621	rs71454237		0.89–0.89	0.89
94	12:93962959:93998321	rs9634212		0.90–0.97	0.93
95	12:114455843:115753430	rs10850315		0.99–1.00	1.00
96	12:133049093:133164690	rs7398059	*CCDC92*, *FBRSL1*	0.84–0.96	0.91
97	12:24452007:24920794	rs11047527	*LINC00477*	0.98–0.98	0.98
98	12:124763774:124823437	rs2272368	*CCDC92*, *NCOR2*	0.95–0.95	0.95
99	12:120624085:120659997	rs12308065	*GCN1*, *PXN*	0.84–0.96	0.90
100	12:109888779:111883779	rs11065836	*CUX2*	0.96–0.96	0.96
101	12:114455843:115903273	rs7964303	*TBX5*	0.99–1.00	1.00
102	13:113818708:113975290	rs2316443	*CUL4A*, *PCID2*, *PROZ*	0.82–0.82	0.82
103	13:111297611:111407858	rs9521916		0.83–0.95	0.93
104	13:21935210:22321988	rs12428749	*MICU2*, *ZDHHC20*, *VPS13C*	0.87–0.87	0.87
105	13:74504415:74546222	rs9573330	*KLF12*	0.94–0.94	0.94
106	14:102534498:102765331	rs12883091	*WDR20*, *MOK*	0.81–0.83	0.82
107	14:23773253:23897507	rs422068	*MYH6*, *MYH7*	0.99–1.00	1.00
108	14:95981610:95990611	rs179150, rs179152	*SYNE3*	0.91–0.96	0.94
109	14:85775977:85858447	rs6574818		0.81–0.81	0.81
110	14:101996529:101999598	rs11160652		0.99–0.99	0.99
111	15:70450177:70464744	rs4777245	*TLE3*	0.89–0.96	0.92
112	15:41892061:99321226	rs28444909, rs6598541	*MAPKBP1*, *IGF1R*, *PLA2G4B*	0.81–0.99	0.92
113	15:62131989:62381630	rs3784633		0.81–0.84	0.83
114	15:73342781:73687693	rs7172796	*HCN4*, *NEO1*	0.93–0.93	0.93
115	15:80978266:81077104	rs2627313	*ABHD17C*	0.92–0.94	0.93
116	16:53797908:53848561	rs9941349	*FTO*	0.82–0.88	0.85
117	16:30122181:30164488	rs7201780		0.83–0.83	0.83
118	16:28826049:29008079	rs28888764		0.96–0.96	0.96
119	16:3810986:3960458	rs2540039	*CREBBP*	0.98–0.98	0.98
120	16:15901583:16152940	rs9284324		0.98–0.98	0.98
121	16:64892558:64899744	rs9924120		0.89–0.89	0.89
122	17:37389409:38215495	rs7359623	*GSDMB*, *MED1*	0.92–0.92	0.92
123	17:68260211:68608228	rs1396517	*TYRO3*	0.85–0.95	0.92
124	17:61664740:62017421	rs2320125	*CD79B*	0.88–0.88	0.88
125	17:3857883:4272282	rs79366918	*ATP2A3*	0.96–0.96	0.96
126	17:76661207:76833916	rs17657522	*MICAL3*, *CYTH1*, *USP36*	0.83–0.91	0.89
127	17:29166302:29263700	rs11867227	*ADAP2*, *TEFM*	0.83–0.83	0.83
128	17:43463493:45281257	rs242557	*MAPT*	0.99–1.00	1.00
129	17:17107740:17140900	rs4985750	*FLCN*, *MPRIP*, *PLD6*	0.97–0.97	0.97
130	18:77156103:77162816	rs56376587	*NFATC1*	0.99–1.00	1.00
131	19:41885151:41946095	rs11667908	*EXOSC5*, *TMEM91*, *B3GNT8*	0.86–0.88	0.87
132	19:2160529:2232049	rs740404	*AP3D1*, *PLEKHJ1*, *MYH11*	0.89–0.89	0.89
133	19:5092931:5192090	rs2620855	*KDM4B*, *PTPRS*	0.94–0.94	0.94
134	20:36807478:36897290	rs3746471	*TGM2*, *KIAA1755*	0.94–0.97	0.95
135	20:61141981:61213252	rs6089752	*MIR1-1HG*	0.93–0.94	0.94
136	22:18281629:18294697	rs9605408		0.94–0.98	0.96
137	22:21911220:22226711	rs12484001	*UBE2L3*, *YDJC*	0.86–0.86	0.86

Table 1 displays 137 distinct colocalized regions consolidated from the 179 colocalized loci between AF and LE8 components, along with the mapped candidate genes within each region. The genomic positions are based on the GRCh38/hg38 genome build, and novel genes are underlined. The colocalization posterior probability (*PPH*_4_) represents the posterior probability of hypothesis 4 (H_4_), indicating the two traits share the same causal variant(s) at the locus. The 156 lead SNPs are considered significant evidence of colocalization with *PPH*_4_ > 0.8. For each region, the “Range of *PPH*_4_” indicates the minimum and maximum values observed for its shared causal variant(s) and the mean values are presented in the “Average of *PPH*_4_”.

**Table 2:** Drug target enrichment of candidate genes.

ATC Group	Drug Name	*OR*	*p* Value	Target Gene and Drug Names
S02	Otologicals *	35.63	2.37 × 10^−4^	*NR3C1* (hydrocortisone, prednisolone, dexamethasone, betamethasone, fluocinolone acetonide), *SCN10A* (lidocaine, cinchocaine), *SCN5A* (lidocaine, cocaine, cinchocaine)
C05	Vasoprotectives	12.39	3.12 × 10^−3^	*NR3C1* (hydrocortisone, prednisolone, betamethasone, fluorometholone, dexamethasone, fluocinolone acetonide, fluocinonide, triamcinolone), *SCN10A* (lidocaine, tetracaine, benzocaine, cinchocaine, procaine), *SCN5A* (lidocaine, cinchocaine)
D04	Antipruritic, including antihistamines, anesthetics, etc.	26.61	4.55 × 10^−3^	*SCN10A* (lidocaine, cinchocaine, oxybuprocaine, benzocaine, tetracaine), *SCN5A* (lidocaine, cinchocaine)
R02	Throat preparations	13.05	2.74 × 10^−3^	*KCNN3* (dequalinium), *SCN10A* (benzocaine, lidocaine, dyclonine), *SCN5A* (lidocaine, cocaine, dyclonine)

Table 2 shows the drug target enrichment for candidate genes between AF and LE8 components by the ATC tests of GREP. The asterisks (*) next to the Drug name indicate statistical significance at the Bonferroni-corrected level (*p* < 5.56 × 10^−4^).

## Data Availability

Our data availability statement conforms to the category of “Data available in a publicly accessible repository”. The original data presented in the study are openly available. All data sources and their corresponding URLs used in this study are provided in Table S1.

## Supplementary Materials

The following supporting information can be downloaded at: https://www.mdpi.com/article/10.3390/biomedicines14061179/s1, Figures S1–S30: Conditional Manhattan plot for AF and LE8 components; Table S1: Description of the GWAS summary data sources; Table S2: Pleiotropic SNPs and loci of cFDR analysis; Table S3: Functional mapped genes and prioritization; Table S4: Pathways Enrichment of Candidate genes; Table S5: Pathways Enrichment of novel AF-associated genes; Table S6: Pathways Enrichment of candidate genes colocalized with AF and BP; Table S7: Pathways Enrichment of candidate genes colocalized with AF and SBP; Table S8: Pathways Enrichment of candidate genes colocalized with AF and DBP; Table S9: Pathways Enrichment of candidate genes colocalized with AF and PP.

## Abbreviations

The following abbreviations are used in this manuscript:

AF	Atrial fibrillation
LE8	Life’s essential 8
GWAS	Genome-wide association study
SNP	Single-nucleotide polymorphism
BP	Blood pressure
LIP	Blood lipid
OBS	Obesity
GLU	Glucose
DIET	Dietary
SMK	Smoking
SLP	Sleeping
PA	Physical Activity
SBP	Systolic Blood pressure
DBP	Diastolic Blood pressure
PP	Pulse pressure
TG	Triglycerides
TC	Total cholesterol
LDLC	Low-density lipoprotein cholesterol
NHDLC	Non-high-density lipoprotein cholesterol
HDLC	High-density lipoprotein cholesterol
BMI	Body mass index
WHR	Waist-to-hip ratio
WHRadBMI	Waist-to-hip ratio adjusted for BMI
T2D	Type 2 diabetes
DIEThf	Healthy food consumption
DIETohd	Overall healthy diet
DIETpc1–5	Principal component-derived dietary pattern 1–5
SMKinit	Smoking initiation status
SMKage	Age of smoking initiation
SMKcigday	Cigarettes per Day
SMKces	Smoking cessation status
SLPdur	Sleep duration
SLPshort	Short sleep duration
SLPlong	Long sleep duration
PAint	Moderate-to-vigorous intensity physical activity during leisure time
PAlst	Leisure screen time
PAsedcm	Sedentary commuting
PAsedwk	Sedentary behavior at work
r_g_	Genetic correlation
LDSC	Linkage disequilibrium score
condFDR	Conditional false discovery rate
*PPH*_4_	Posterior probability of hypothesis 4
H_4_	Hypothesis 4
H_0_	Hypothesis 0
VEP	Ensembl Variant Effect Predictor
V2G	Variant-to-Gene
PoPS	Polygenic priority score
SmultiXcan	Summary-MultiXcan
HuGE	Human Genetic Evidence
L2G	Locus-to-Gene
CVDKP	Cardiovascular Disease Knowledge Portal
FUMA	Functional mapping and annotation of genetic associations
GO:BP	Gene Ontology Biological Process
GO:MF	Gene Ontology Molecular Function
GO:CC	Gene Ontology Cellular Component
ATC	Anatomical Therapeutic Chemical Classification
GREP	Genome for REPositioning drugs
PRS	Polygenic Risk Score
CVDs	Cardiovascular diseases
RHD	Rheumatic heart diseases
HD	Hypertensive diseases
IHD	Ischemic heart diseases
CP	cor pulmonale
PD	Pericardial diseases
VHD	Valvular heart diseases
MD	Myocardial diseases
AR	Arrhythmia
HF	Heart failure
OHD	Other heart diseases
CED	Cerebrovascular disease
AAD	Diseases of arteries, arterioles and capillaries
VLD	Diseases of veins, lymphatic vessels and lymph nodes
OCD	Other and unspecified disorders of the circulatory system
*OR*	Odds ratio
*HR*	Hazard ratio
*CI*	Confidence interval
