# Supplementary material for: Uncovering Novel Atrial Fibrillation Genetics Through Pleiotropic Overlap with Life’s Essential 8
Source: Biomedicines. 2026 May 22;14(6):1179. doi: 10.3390/biomedicines14061179 (PMC13296989; doi:10.3390/biomedicines14061179)
Supplement: Supplementary file 1 [file biomedicines-14-01179-s001.zip › Supplementary Figures 1-30.pdf]

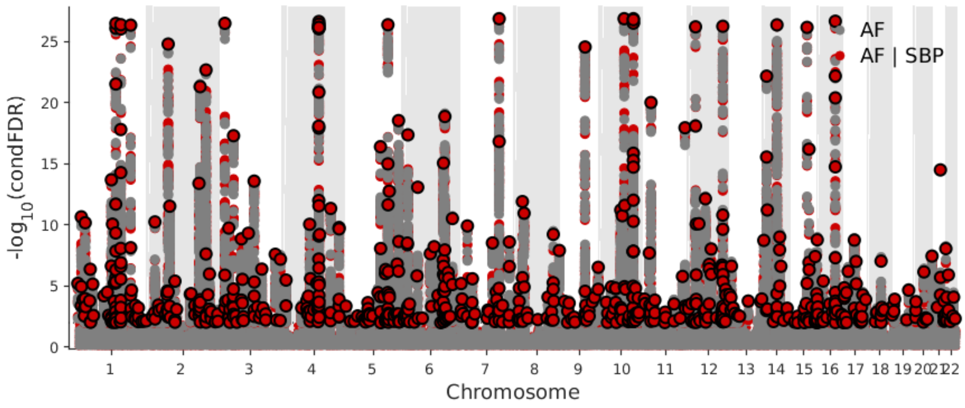

Supplementary Figure S1: Conditional Manhattan plot for AF and SBP

The conditional Manhattan plot displays the -log10(condFDR) for SNPs on the y-axis, with genomic position across chromosomes (1-22) on the x-axis. Gray dots represent SNPs that were involved in the original AF GWAS. Red dots indicate SNPs that remained significant (condFDR < 0.01) after conditioning on SBP. Abbreviations: Atrial fibrillation, AF; systolic blood pressure, SBP; Conditional false discovery rate, condFDR.

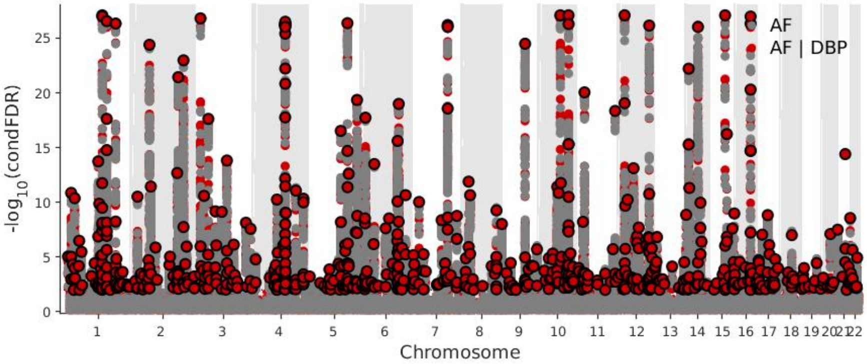

Supplementary Figure S2: Conditional Manhattan plot for AF and DBP

The conditional Manhattan plot displays the -log10(condFDR) for SNPs on the y-axis, with genomic position across all chromosomes (1-22) on the x-axis. Gray dots represent SNPs that were involved in the original AF GWAS. Red dots indicate SNPs that remained significant (condFDR < 0.01) after conditioning on DBP. Abbreviations: Atrial fibrillation, AF; diastolic blood pressure, DBP; Conditional false discovery rate, condFDR.

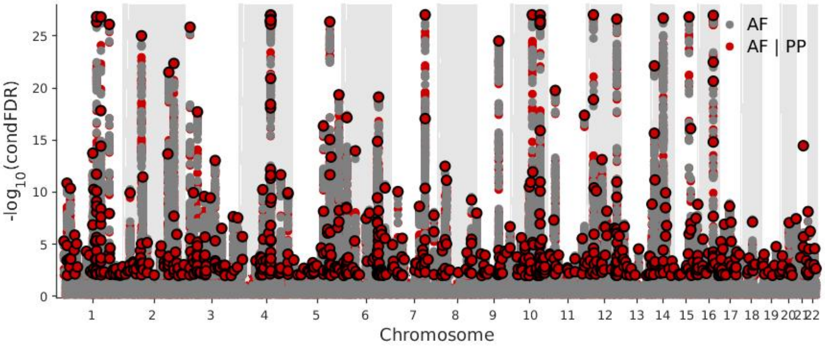

Supplementary Figure S3: Conditional Manhattan plot for AF and PP

The conditional Manhattan plot displays the -log10(condFDR) for SNPs on the y-axis, with genomic position across chromosomes (1-22) on the x-axis. Gray dots represent SNPs that were involved in the original AF GWAS. Red dots indicate SNPs that remained significant (condFDR < 0.01) after conditioning on PP. Abbreviations: Atrial fibrillation, AF; pulse pressure, PP; Conditional false discovery rate, condFDR.

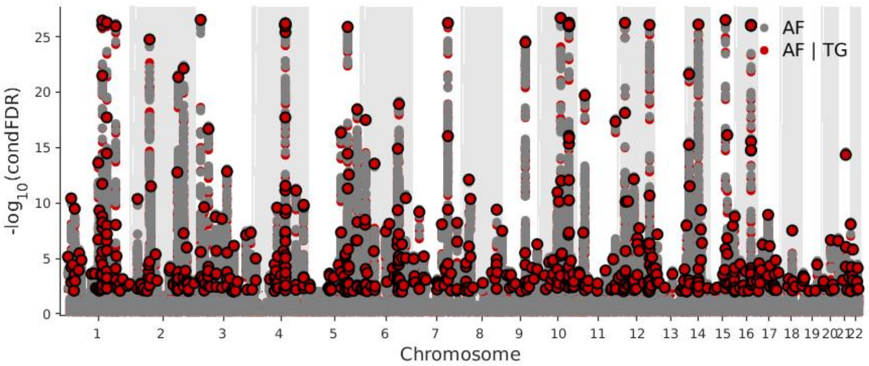

Supplementary Figure S4: Conditional Manhattan plot for AF and TG

The conditional Manhattan plot displays the -log10(condFDR) for SNPs on the y-axis, with genomic position across chromosomes (1-22) on the x-axis. Gray dots represent SNPs that were involved in the original AF GWAS. Red dots indicate SNPs that remained significant (condFDR < 0.01) after conditioning on TG. Abbreviations: Atrial fibrillation, AF; triglycerides, TG; Conditional false discovery rate, condFDR.

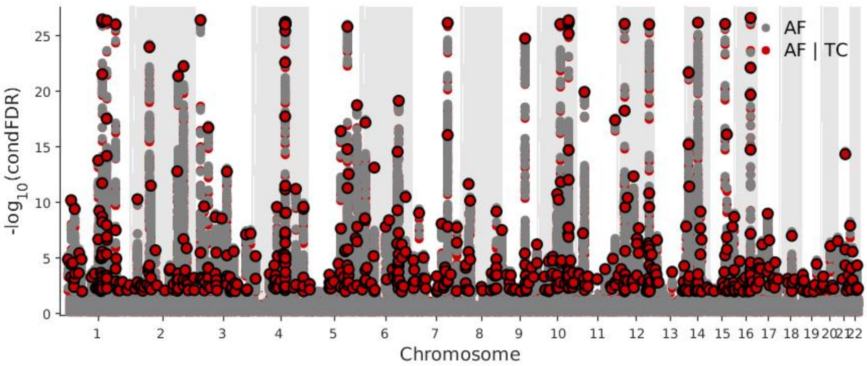

Supplementary Figure S5: Conditional Manhattan plot for AF and TC

The conditional Manhattan plot displays the -log10(condFDR) for SNPs on the y-axis, with genomic position across chromosomes (1-22) on the x-axis. Gray dots represent SNPs that were involved in the original AF GWAS. Red dots indicate SNPs that remained significant (condFDR < 0.01) after conditioning on TC. Abbreviations: Atrial fibrillation, AF; total cholesterol, TC; Conditional false discovery rate, condFDR.

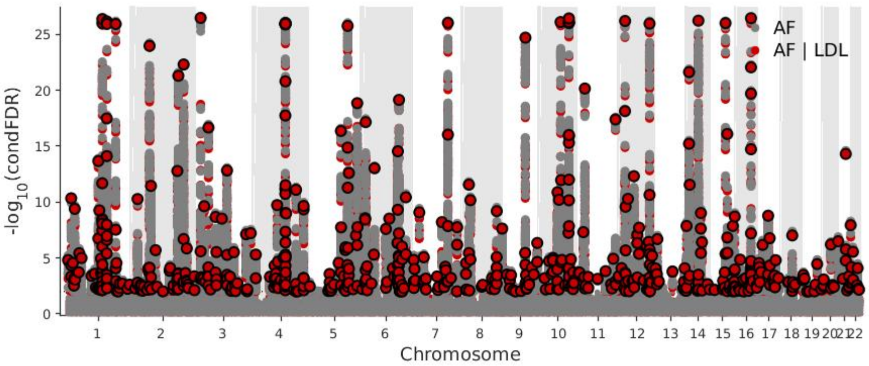

Supplementary Figure S6: Conditional Manhattan plot for AF and LDLC

The conditional Manhattan plot displays the -log10(condFDR) for SNPs on the y-axis, with genomic position across chromosomes (1-22) on the x-axis. Gray dots represent SNPs that were involved in the original AF GWAS. Red dots indicate SNPs that remained significant (condFDR < 0.01) after conditioning on LDLC. Abbreviations: Atrial fibrillation, AF; low-density lipoprotein cholesterol, LDLC; Conditional false discovery rate, condFDR.

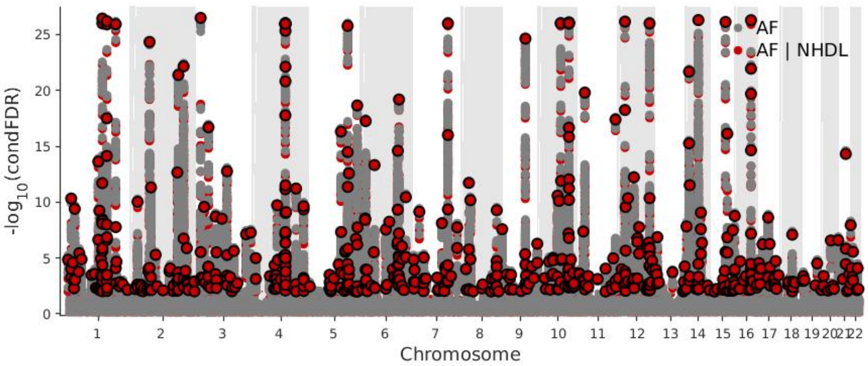

Supplementary Figure S7: Conditional Manhattan plot for AF and NHDLC

The conditional Manhattan plot displays the -log10(condFDR) for SNPs on the y-axis, with genomic position across chromosomes (1-22) on the x-axis. Gray dots represent SNPs that were involved in the original AF GWAS. Red dots indicate SNPs that remained significant (condFDR < 0.01) after conditioning on NHDLC. Abbreviations: Atrial fibrillation, AF; non-high-density lipoprotein cholesterol, NHDLC; Conditional false discovery rate, condFDR.

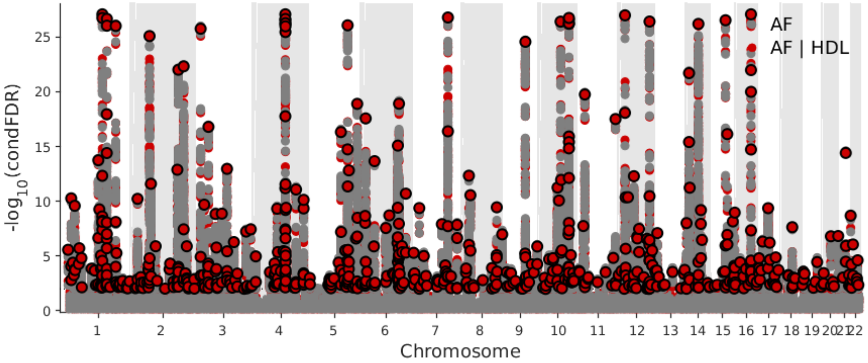

Supplementary Figure S8: Conditional Manhattan plot for AF and HDLC

The conditional Manhattan plot displays the -log10(condFDR) for SNPs on the y-axis, with genomic position across chromosomes (1-22) on the x-axis. Gray dots represent SNPs that were involved in the original AF GWAS. Red dots indicate SNPs that remained significant (condFDR < 0.01) after conditioning on HDLC. Abbreviations: Atrial fibrillation, AF; high-density lipoprotein cholesterol, HDLC; Conditional false discovery rate, condFDR.

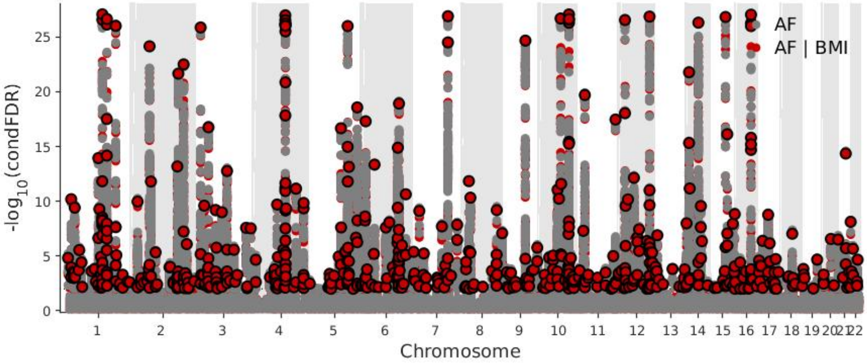

Supplementary Figure S9: Conditional Manhattan plot for AF and BMI

The conditional Manhattan plot displays the -log10(condFDR) for SNPs on the y-axis, with genomic position across chromosomes (1-22) on the x-axis. Gray dots represent SNPs that were involved in the original AF GWAS. Red dots indicate SNPs that remained significant (condFDR < 0.01) after conditioning on BMI. Abbreviations: Atrial fibrillation, AF; body mass index, BMI; Conditional false discovery rate, condFDR.

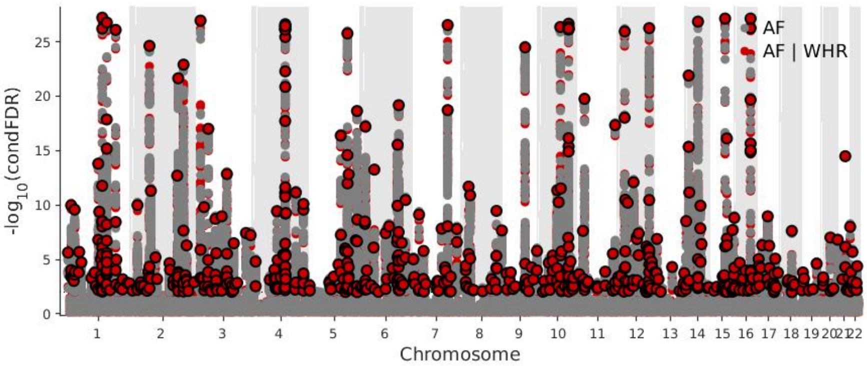

Supplementary Figure S10: Conditional Manhattan plot for AF and WHR

The conditional Manhattan plot displays the -log10(condFDR) for SNPs on the y-axis, with genomic position across chromosomes (1-22) on the x-axis. Gray dots represent SNPs that were involved in the original AF GWAS. Red dots indicate SNPs that remained significant (condFDR < 0.01) after conditioning on WHR. Abbreviations: Atrial fibrillation, AF; waist-to-hip ratio, WHR; Conditional false discovery rate, condFDR.

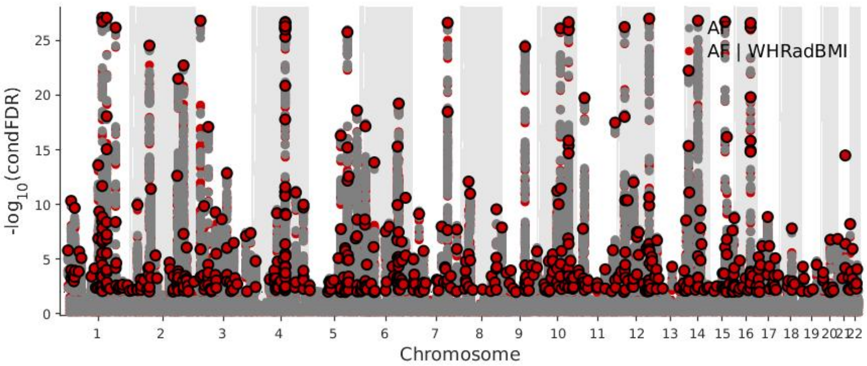

Supplementary Figure S11: Conditional Manhattan plot for AF and WHRadBMI

The conditional Manhattan plot displays the -log10(condFDR) for SNPs on the y-axis, with genomic position across chromosomes (1-22) on the x-axis. Gray dots represent SNPs that were involved in the original AF GWAS. Red dots indicate SNPs that remained significant (condFDR < 0.01) after conditioning on WHRadBMI. Abbreviations: Atrial fibrillation, AF; waist-to-hip ratio adjusted for body mass index, WHRadBMI; Conditional false discovery rate, condFDR.

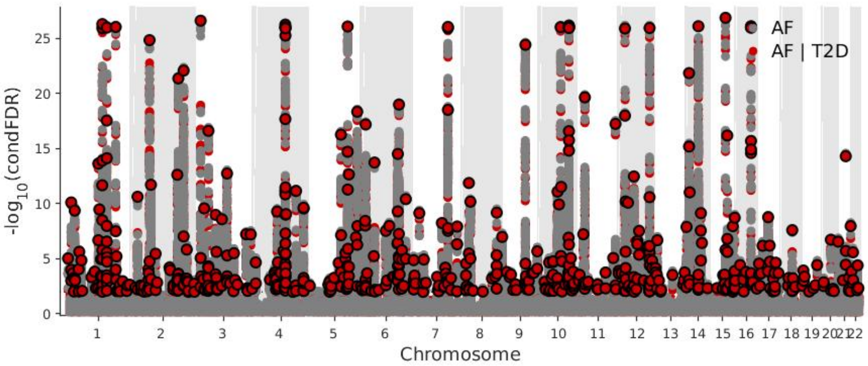

Supplementary Figure S12: Conditional Manhattan plot for AF and T2D

The conditional Manhattan plot displays the -log10(condFDR) for SNPs on the y-axis, with genomic position across chromosomes (1-22) on the x-axis. Gray dots represent SNPs that were involved in the original AF GWAS. Red dots indicate SNPs that remained significant (condFDR < 0.01) after conditioning on T2D. Abbreviations: Atrial fibrillation, AF; type 2 diabetes, T2D; Conditional false discovery rate, condFDR.

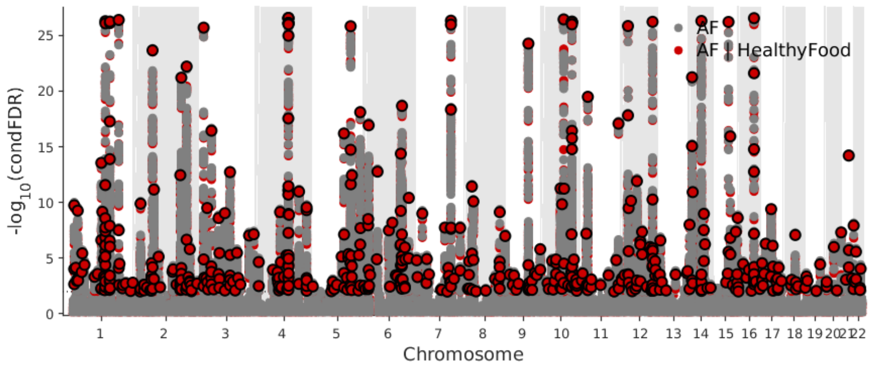

Supplementary Figure S13: Conditional Manhattan plot for AF and DIETHf

The conditional Manhattan plot displays the -log10(condFDR) for SNPs on the y-axis, with genomic position across chromosomes (1-22) on the x-axis. Gray dots represent SNPs that were involved in the original AF GWAS. Red dots indicate SNPs that remained significant (condFDR < 0.01) after conditioning on DIETHf. healthy food consumption, DIETHf; Conditional false discovery rate, condFDR.

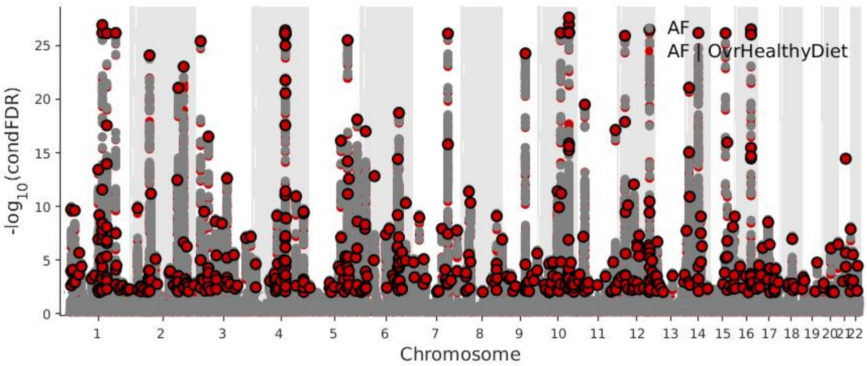

Supplementary Figure S14: Conditional Manhattan plot for AF and DIETohd

The conditional Manhattan plot displays the -log10(condFDR) for SNPs on the y-axis, with genomic position across chromosomes (1-22) on the x-axis. Gray dots represent SNPs that were involved in the original AF GWAS. Red dots indicate SNPs that remained significant (condFDR < 0.01) after conditioning on DIETohd. Abbreviations: Atrial fibrillation, AF; overall healthy diet,DIETohd; Conditional false discovery rate, condFDR.

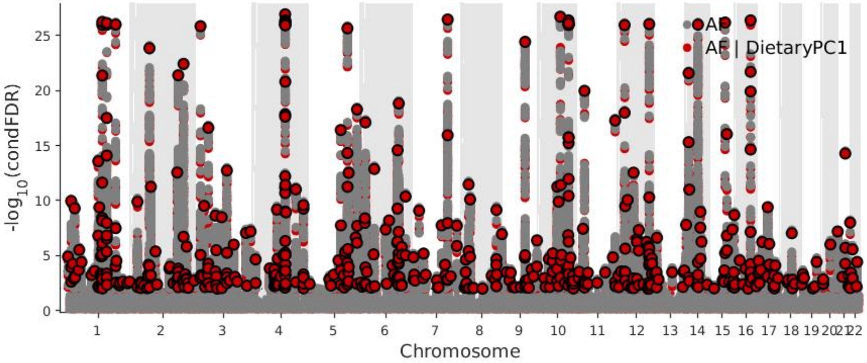

Supplementary Figure S15: Conditional Manhattan plot for AF and DIETpc1

The conditional Manhattan plot displays the -log10(condFDR) for SNPs on the y-axis, with genomic position across chromosomes (1-22) on the x-axis. Gray dots represent SNPs that were involved in the original AF GWAS. Red dots indicate SNPs that remained significant (condFDR < 0.01) after conditioning on the DIETpc1. Abbreviations: Atrial fibrillation, AF; principal component-derived dietary pattern 1, DIETpc1; Conditional false discovery rate, condFDR.

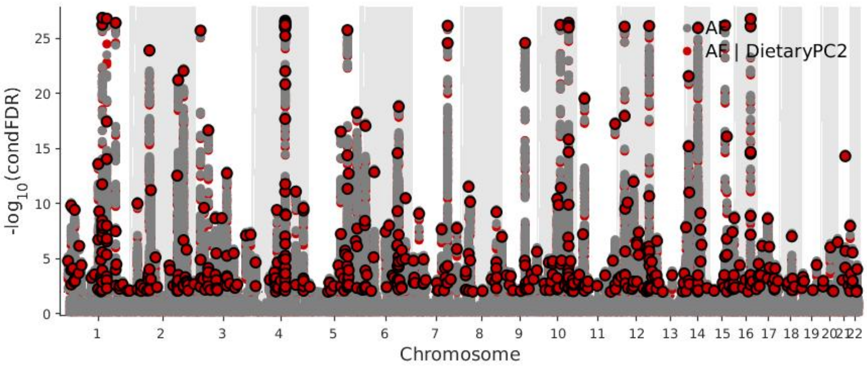

Supplementary Figure S16: Conditional Manhattan plot for AF and DIETpc2

The conditional Manhattan plot displays the -log10(condFDR) for SNPs on the y-axis, with genomic position across chromosomes (1-22) on the x-axis. Gray dots represent SNPs that were involved in the original AF GWAS. Red dots indicate SNPs that remained significant (condFDR < 0.01) after conditioning on the DIETpc2. Abbreviations: Atrial fibrillation, AF; principal component-derived dietary pattern 2, DIETpc2; Conditional false discovery rate, condFDR.

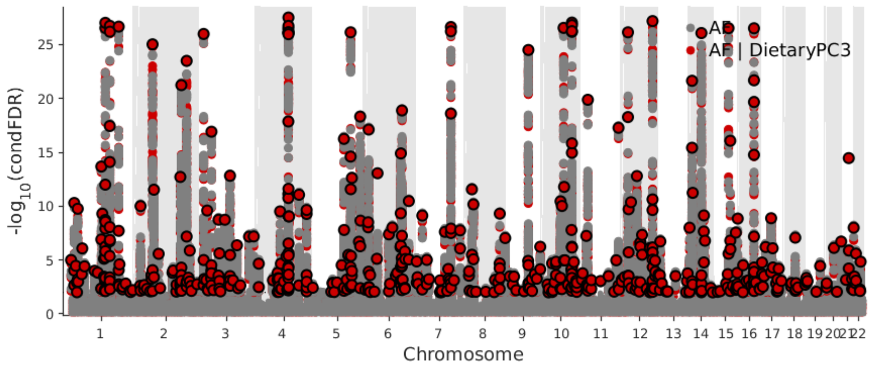

Supplementary Figure S17: Conditional Manhattan plot for AF and DIETpc3

The conditional Manhattan plot displays the -log10(condFDR) for SNPs on the y-axis, with genomic position across chromosomes (1-22) on the x-axis. Gray dots represent SNPs that were involved in the original AF GWAS. Red dots indicate SNPs that remained significant (condFDR < 0.01) after conditioning on the DIETpc3. Abbreviations: Atrial fibrillation, AF; principal component-derived dietary pattern 3, DIETpc3; Conditional false discovery rate, condFDR.

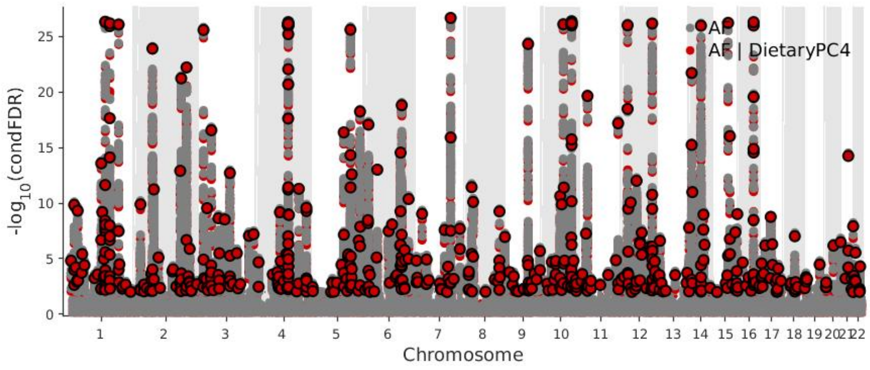

Supplementary Figure S18: Conditional Manhattan plot for AF and DIETpc4

The conditional Manhattan plot displays the -log10(condFDR) for SNPs on the y-axis, with genomic position across chromosomes (1-22) on the x-axis. Gray dots represent SNPs that were involved in the original AF GWAS. Red dots indicate SNPs that remained significant (condFDR < 0.01) after conditioning on the DIETpc4. Abbreviations: Atrial fibrillation, AF; principal component-derived dietary pattern 4, DIETpc4; Conditional false discovery rate, condFDR.

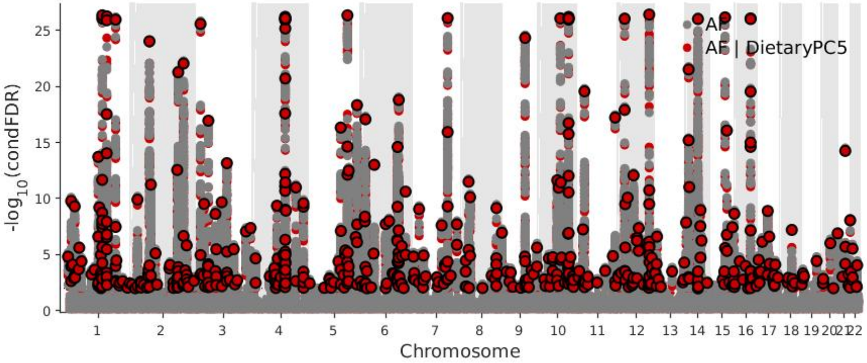

Supplementary Figure S19: Conditional Manhattan plot for AF and DIETpc5

The conditional Manhattan plot displays the -log10(condFDR) for SNPs on the y-axis, with genomic position across chromosomes (1-22) on the x-axis. Gray dots represent SNPs that were involved in the original AF GWAS. Red dots indicate SNPs that remained significant (condFDR < 0.01) after conditioning on the DIETpc5. Abbreviations: Atrial fibrillation, AF; principal component-derived dietary pattern 5, DIETpc5; Conditional false discovery rate, condFDR.

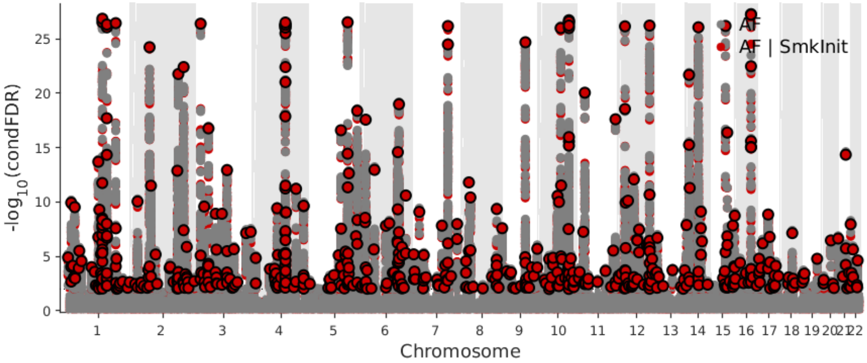

Supplementary Figure S20: Conditional Manhattan plot for AF and SMKinit

The conditional Manhattan plot displays the -log10(condFDR) for SNPs on the y-axis, with genomic position across chromosomes (1-22) on the x-axis. Gray dots represent SNPs that were involved in the original AF GWAS. Red dots indicate SNPs that remained significant (condFDR < 0.01) after conditioning on the SMKinit. Abbreviations: Atrial fibrillation, AF; smoking initiation status, SMKinit; Conditional false discovery rate, condFDR.

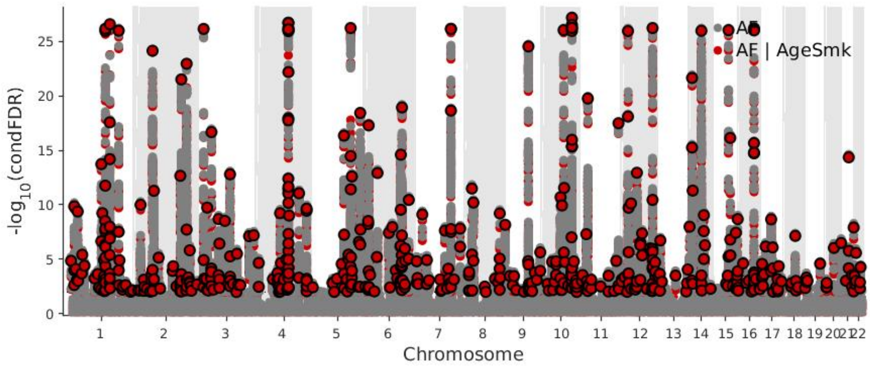

Supplementary Figure S21: Conditional Manhattan plot for AF and SMKage

The conditional Manhattan plot displays the -log10(condFDR) for SNPs on the y-axis, with genomic position across chromosomes (1-22) on the x-axis. Gray dots represent SNPs that were involved in the original AF GWAS. Red dots indicate SNPs that remained significant (condFDR < 0.01) after conditioning on the age of SMKage. Abbreviations: Atrial fibrillation, AF; smoking initiation, SMKage; Conditional false discovery rate, condFDR.

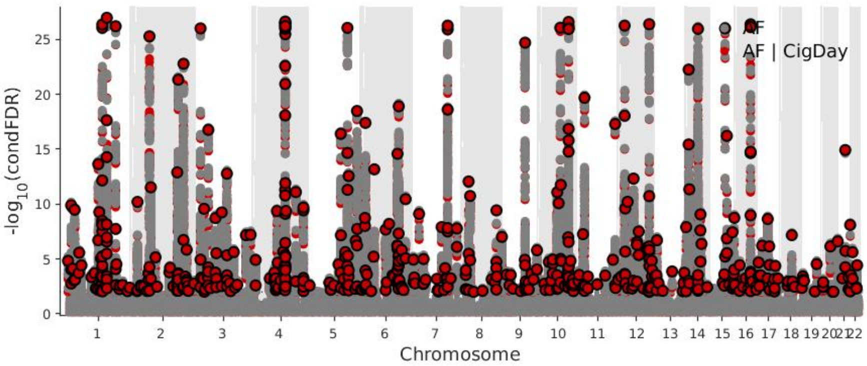

Supplementary Figure S22: Conditional Manhattan plot for AF and SMKcig

The conditional Manhattan plot displays the -log10(condFDR) for SNPs on the y-axis, with genomic position across chromosomes (1-22) on the x-axis. Gray dots represent SNPs that were involved in the original AF GWAS. Red dots indicate SNPs that remained significant (condFDR < 0.01) after conditioning on the SMKcig. Abbreviations: Atrial fibrillation, AF; cigarettes per Day, SMKcig; Conditional false discovery rate, condFDR.

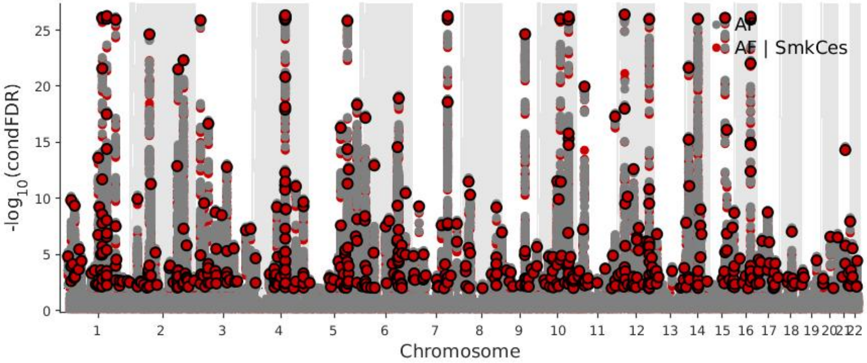

Supplementary Figure S23: Conditional Manhattan plot for AF and SMKces

The conditional Manhattan plot displays the -log10(condFDR) for SNPs on the y-axis, with genomic position across chromosomes (1-22) on the x-axis. Gray dots represent SNPs that were involved in the original AF GWAS. Red dots indicate SNPs that remained significant (condFDR < 0.01) after conditioning on the SMKces. Abbreviations: Atrial fibrillation, AF; smoking cessation status, SMKces; Conditional false discovery rate, condFDR.

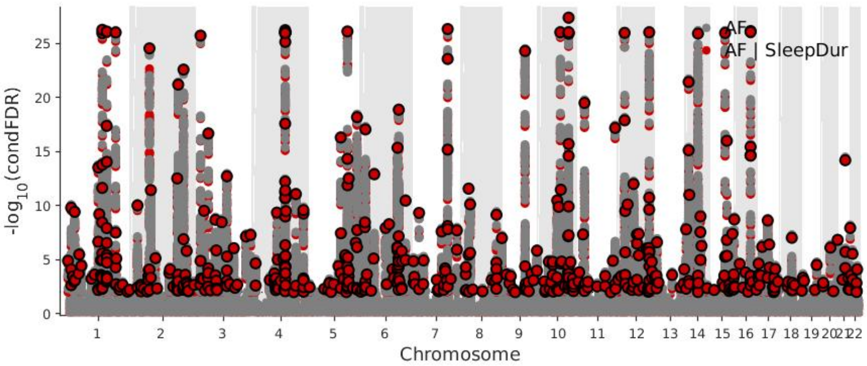

Supplementary Figure S24: Conditional Manhattan plot for AF and SLPdur

The conditional Manhattan plot displays the -log10(condFDR) for SNPs on the y-axis, with genomic position across chromosomes (1-22) on the x-axis. Gray dots represent SNPs that were involved in the original AF GWAS. Red dots indicate SNPs that remained significant (condFDR < 0.01) after conditioning on the SLPdur. Abbreviations: Atrial fibrillation, AF; sleep duration, SLPdur; Conditional false discovery rate, condFDR.

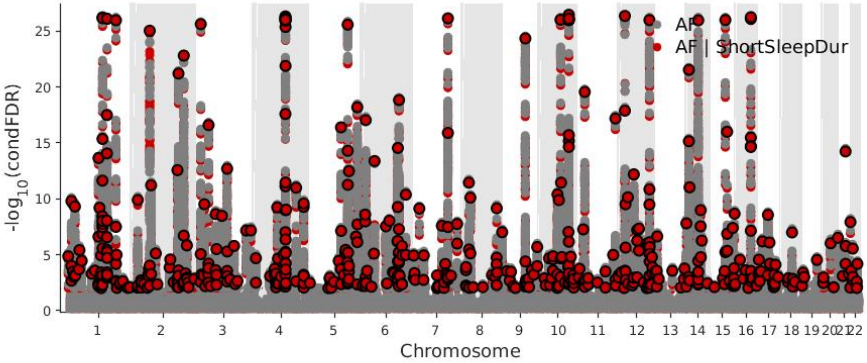

Supplementary Figure S25: Conditional Manhattan plot for AF and SLPshort

The conditional Manhattan plot displays the -log10(condFDR) for SNPs on the y-axis, with genomic position across chromosomes (1-22) on the x-axis. Gray dots represent SNPs that were involved in the original AF GWAS. Red dots indicate SNPs that remained significant (condFDR < 0.01) after conditioning on the SLPshort. Abbreviations: Atrial fibrillation, AF; short sleep duration, SLPshort; Conditional false discovery rate, condFDR.

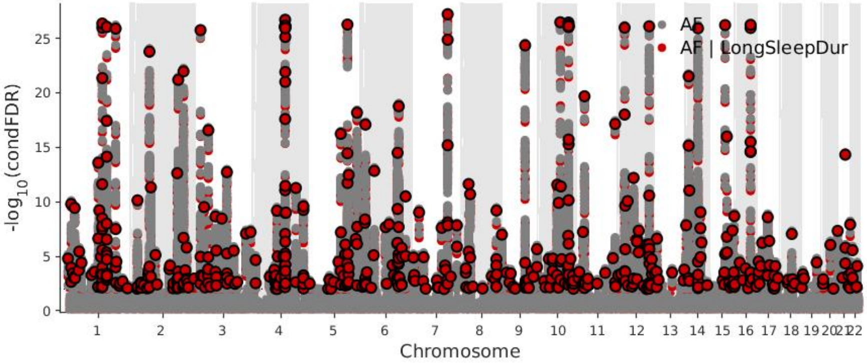

Supplementary Figure S26: Conditional Manhattan plot for AF and SLPlong

The conditional Manhattan plot displays the -log10(condFDR) for SNPs on the y-axis, with genomic position across chromosomes (1-22) on the x-axis. Gray dots represent SNPs that were involved in the original AF GWAS. Red dots indicate SNPs that remained significant (condFDR < 0.01) after conditioning on the SLPlong. Abbreviations: Atrial fibrillation, AF; short sleep duration, SLPlong; Conditional false discovery rate, condFDR.

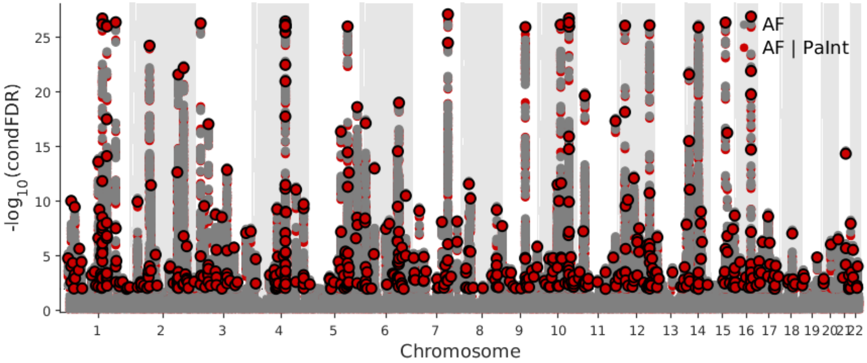

Supplementary Figure S27: Conditional Manhattan plot for AF and PAint

The conditional Manhattan plot displays the -log10(condFDR) for SNPs on the y-axis, with genomic position across chromosomes (1-22) on the x-axis. Gray dots represent SNPs that were involved in the original AF GWAS. Red dots indicate SNPs that remained significant (condFDR < 0.01) after conditioning on the PAint. Abbreviations: Atrial fibrillation, AF; moderate-to-vigorous intensity physical activity during leisure time, PAinit; Conditional false discovery rate, condFDR.

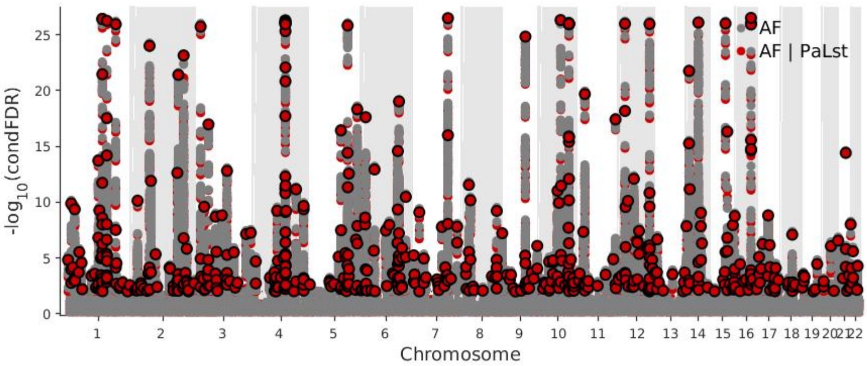

Supplementary Figure S28: Conditional Manhattan plot for AF and PAlst

The conditional Manhattan plot displays the -log10(condFDR) for SNPs on the y-axis, with genomic position across chromosomes (1-22) on the x-axis. Gray dots represent SNPs that were involved in the original AF GWAS. Red dots indicate SNPs that remained significant (condFDR < 0.01) after conditioning on the PAlst. Abbreviations: Atrial fibrillation, AF; Leisure screen time, PAlst; Conditional false discovery rate, condFDR.

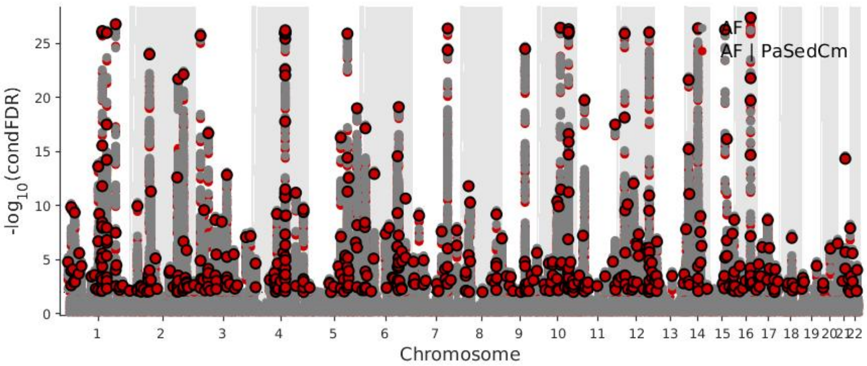

Supplementary Figure S29: Conditional Manhattan plot for AF and PAsedcm

The conditional Manhattan plot displays the -log10(condFDR) for SNPs on the y-axis, with genomic position across chromosomes (1-22) on the x-axis. Gray dots represent SNPs that were involved in the original AF GWAS. Red dots indicate SNPs that remained significant (condFDR < 0.01) after conditioning on the PAsedcm. Abbreviations: Atrial fibrillation, AF; sedentary commuting, PAsedcm; Conditional false discovery rate, condFDR.

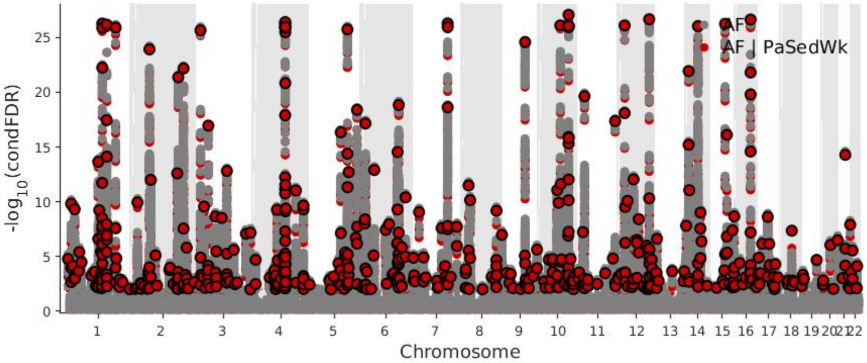

Supplementary Figure S30: Conditional Manhattan plot for AF and PAsedwk

The conditional Manhattan plot displays the -log10(condFDR) for SNPs on the y-axis, with genomic position across chromosomes (1-22) on the x-axis. Gray dots represent SNPs that were involved in the original AF GWAS. Red dots indicate SNPs that remained significant (condFDR < 0.01) after conditioning on the PAsedwk. Abbreviations: Atrial fibrillation, AF; sedentary behavior at work, PAsedwk; Conditional false discovery rate, condFDR.
